# The impact of a training programme incorporating the conceptual framework of the International Classification of Functioning (ICF) on behaviour regarding interprofessional practice in Rwandan health professionals: A cluster randomized control trial

**DOI:** 10.1371/journal.pone.0226247

**Published:** 2020-02-07

**Authors:** Jean Baptiste Sagahutu, Jeanne Kagwiza, Francois Cilliers, Jennifer Jelsma

**Affiliations:** 1 University of Rwanda, College of Medicine and Health Sciences, Kigali, Rwanda; 2 University of Cape Town, Cape Town, South Africa; IRCCS E. Medea, ITALY

## Abstract

**Background:**

Appropriate collaboration between health professionals (HP) can reduce medical errors, enhance the spread of critical information, and assist in interpretation of health information resulting in improved patient care. The International Classification of Functioning, Disability and Health (ICF) may provide a useful conceptual framework to facilitate better interprofessional practice.

**Purpose:**

To determine whether a training programme based on the ICF framework resulted in improved interprofessional behaviour among HPs in Rwanda.

**Methodology:**

A cluster randomised control trial was used. Four district hospitals were randomly allocated to receive either a day’s training in interprofessional practice based on the ICF framework (experimental) or a short talk and a booklet on the topic (control). A total of 203 participants included medical doctors, nurses, and other HPs took part in this study. Simple random sampling was used to select the hospital records of 200 patients discharged from relevant wards at both the experimental and control hospitals at baseline and at two, four and six months after training (800 patients’ records from each group). A self-designed checklist has undergone some validation and was based on the ICF conceptual model was used to audit the quality of information included in the patients’ records. Ethical approval was obtained from the relevant authorities.

**Results:**

The demographic and medical profile of the patients in the two sets was equivalent. An ANOVA and post-hoc Tukey test indicated the mean number of items correctly filled in was not significant at baseline (p = 0.424) but the difference was significant (p < .001) for the post-intervention scores at two, four and six months. The control group scores did not improve over time. The improved behavior was still evident at six months although it had begun to decay.

**Conclusion:**

Behaviour change as evidenced by more comprehensive recording of patient management can result from a well-structured training programme. The ICF appeared to provide a common language and facilitate HPs interaction and patient management plans.

**Implication:**

The ICF provided an effective conceptual framework to structure the content of the training and the audit tool. It is recommended that the framework be used to facilitate interprofessional education and practice in Rwanda and that the training approach may be applicable to other health care contexts.

## Background

Despite large gains in health status globally, brought about in part by the scientific application of medical science, there are still large inequalities in health and health care provision, both within and between countries. The provision of appropriate health care is primarily the responsibility of health professional (HP) who should be involved in health promotion, prevention, diagnosis and treatment of disease and other health related conditions with the ultimate goal of promoting health outcomes of the individual and the population as a whole [1]. Unfortunately the Lancet Global Independent Commission tasked with reviewing health education concluded that the training and education of HP is producing graduates who are ill-equipped to deal with current health care requirements [2]. Problems identified included (amongst others): poor teamwork, “*persistent gender stratification of professional status” and the “so-called tribalism of the professions—i*.*e*., *the tendency of the various professions to act in isolation from or even in competition with each other*” p.5). In addition a lack of continuity of care was identified as impacting negatively on the health of populations [2]. The Commission identified the need for health care reform driven by transformed HP curricula based on interprofessional and transprofessional education. This would assist in breaking down professional silos while enhancing collaborative and non-hierarchical relationships in effective teams [2].

Another factor obstructing universal access to appropriate health care might be the persistence of the bio-medical model of illness. This model concentrates on the health condition of the patients and treatment is provided within health institutions without adequately addressing the environment of the patient [3]. The bio-psychosocial model, in contrast, recognises that the patient lives within a certain context and that both personal factors and environmental factors should be considered during assessment and management [4]. The need for health care reform (specifically the need for improved interprofessional collaboration), a lack of the continuum of care and over reliance on the medical model of care were noted in Rwanda in a context similar to that of the proposed study. In a study on 500 people living with HIV attending district hospitals in Rwanda, Kagwiza concluded that, as the prevalence of disability was considerable and could not be addressed simply by pharmacological medical management, there was a clear need to promote interprofessional collaborative practice based on a bio-psychosocial approach to reinforce referral within the hospital system [5]. Although the subjects were all people living with HIV, the conclusions are likely to be generalisable to other patient groups.

Interprofessional collaboration occurs when HPs from diverse disciplines and different backgrounds provide patient care collaboratively and work closely with each other and the patient, family and the community to deliver optimum health care [6, 7]. Interprofessional patient oriented care that takes into account all factors which can determine health and functioning of an individual is likely to have the greatest impact on the patient’s quality of life [8]. Therefore, there is a need for an interprofessional framework which should guide communication and collaboration between health care professionals in their clinical settings.

The International Classification of Functioning Disability and Health (ICF) has been suggested as a useful potential framework. The ICF firstly provides a common language for collaborative practice that looks beyond mortality and disease to the impact that health conditions have on peoples’ lives [9]. This is done within a conceptual framework which posits the interaction between health condition, impairments, functional limitation/activity restrictions and environmental and personal factors. The impact of these factors is not necessarily linear, as suggested by the bio-medical model in which the health condition gives rise to impairments and functional limitations. Rather, the relationship is interactive with one component influencing and being in turn influenced by other factors. For example poor living conditions can give rise to health conditions and the impact of these health conditions is then exacerbated by the poor living conditions. However, the application of the ICF is as yet somewhat limited among HPs, especially those who are not part of a rehabilitation team. In addition, there are limited published accounts of training an interprofessional team to adopt ICF in daily practice. This is the case of health care professionals in Rwanda, where the patient assessment and management are oriented towards the profession of the HP and the health condition or impairments of the patients. We hypothesized that the ICF would provide a useful framework within which to structure the assessment of patients and other clients, not only for rehabilitation professionals but for medical practitioners and other HPs who are involved in the care of patients [1].

As recommended by the Lancet, the interprofessional care would result in improved patient outcome [2]. We therefore developed a training programme whose content was based on the ICF and wished to determine whether this training would result in improved behaviour regarding interprofessional practice within Rwandan hospitals. “Improved behaviour” was defined operationally as more comprehensive and holistic reporting by HP in the records of patients admitted to randomly selected district hospitals. We hypothesised that although there would be an initial improvement in behaviour after intervention, this behaviour would decline and possibly be extinct after six months. Finally, it was anticipated that the impact might be different across the different wards, due to the difference in complexity and duration of the conditions. Therefore, the aim of this study was to determine whether a training programme based on the ICF framework resulted in improved interprofessional behaviour among HPs in Rwanda.

## Methods

### Design and sampling

A Cluster Randomised Control Trial (RCT) using a pragmatic study design was carried out to select provinces. Rwanda has 40 district hospitals covering four provinces plus four district hospitals in Kigali city to make a total of 44 district hospitals [10]. In general, all district hospitals in Rwanda are similar in terms of patients and conditions, services, materials and equipment as well as health care personnel. A cluster design is primarily used to avoid contamination between the control and experimental groups when a single setting (such as a ward or hospital) is utilised [11]. Stratified random sampling was used to select the district hospitals within 40 districts of four provinces (North, South, East, and West). Boxes were used to represent the provinces. The names of all hospitals in each province were written on pieces of paper and deposited in the corresponding box representing the province. A blind-folded person randomly picked two hospitals in the Eastern Province and two hospitals in the Northern Province and then, using the same procedure, the same individual chose one of the two hospitals from each box to randomly allocated two hospitals to the experimental arm and the remaining hospital was in the control arm.

Two hospitals were randomly allocated to either receive a day’s training in interprofessional practice using the ICF (experimental hospitals) or a short talk on the topic (control hospitals). A total of 203 HPs who met the inclusion criteria were conveniently selected to participate in this study. Audits of randomly selected patients’ record of recently discharged patients, stratified by ward was done at four time points to determine both immediate and medium term effects of the intervention on behaviour).

A sample of 400 patients’ records (100 from each hospital at every audit) was selected and in all a total of 1600 patients’ records were audited on four occasions (pre-intervention at baseline, and post-intervention at two-month, four-month, and six-month). Records of patients who had been admitted to the surgical, medical and paediatric wards and had stayed for at least five days and records of patients who have been discharged from the above mentioned wards within the two months prior to the date of assessment were included.

### Instrumentation

The intervention was loosely modelled on an original programme offered by the Centre for Health Professions Education, the University of Stellenbosch, South Africa and drew on evidence based principles of learning. The model of the transfer of training process by Grossman and Salas [12] adopted from Baldwin and Ford [13] considered to be the most appropriate for adult learners. This postulates that for the training to be effective in terms of transfer of acquired knowledge, behaviour and attitudes to daily working practice the training has to encompass training characteristics, training design and the work environment. The majority of medical professionals in Rwanda have been trained in and use English in their daily practice. The presentation was thus given in English and, if needed, the researcher used either the local language (Kinyarwanda) or French when greater clarity was needed.

The planned programme was piloted at a fifth district hospital and modified based on the feedback received. The content of the training for the intervention group included sessions on role clarification, patient/client/family/community centred care, team functioning, collaborative leadership, interprofessional communication, and interprofessional conflict resolution. The ICF and its framework was presented as the framework to provide a common language between health care professionals. Small groups then analysed three hospital records to identify the problems that were addressed in the patient records and probable problems that were not addressed paying particular attention to each component of the ICF. The participants were then asked to identify both the facilitators and barriers to implementing holistic health care and interprofessional practice in their hospital and suggest ways to improve practice.

A checklist designed by the author was used to audit patients’ records in behaviour regarding interprofessional practice. The decision was made, based on the several studies reported in the Cochrane Review [14] which used improved documentation as a measure of behavioural change. For the checklist auditing the quality of patients’ records, extensive search was also performed by consulting various databases. The following databases were searched: PubMed (which includes Medline), Cochrane Library, EBSCO, CINAHL Cumulative Index of Nursing and Allied Health, Google Scholar, DIRUM (Database of Instruments for Research use Measurement) and Web of Science. Content validity was assured by a panel of three physiotherapists with higher degrees who had experience with the use of ICF in education, research and practice in South Africa or Canada. Two of these were members of the Functioning and Disability Group of the WHO Family of International Classifications which is the developer and custodian of the ICF. The content validity as established by the panel was excellent (S-CVI = 1.0). Two independent raters audited 30 patient records drawn from the different wards and the Intra-class correlation for absolute agreement between the two independent raters was .885, which indicates excellent agreement.

The Auditing Patient Record checklist is composed of five parts: the patient’s demographic information, comprehensive assessment, holistic intervention, continuum of care, discharge and inter-professional practice. This checklist used the ICF components to determine whether the impact of condition on functioning as well as the impact of environmental factors were recorded in the patient records. Translation of the Auditing Patient Record checklist was not needed because it was utilised by the physiotherapist who had an English educational background.

### Procedure

Within a unitary public health care system such as Rwanda, it is particularly important to obtain support from all relevant authorities, including the Department of Health, before embarking on a health care intervention. After selecting hospitals, all the relevant authorities and institutions were informed of the study in face to face meetings in order to facilitate access to the hospitals. These included the Director General of Clinical and Public Health Services in the Ministry of Health, Hospital Directors, Medical chief of staff and Chief of Nursing.

Stratified random sampling was used to select the patients’ records that met the inclusion criteria to be involved in the study. All eligible patients’ records were given numbers according to their respective wards. The number representing each patient record was written on a slip of paper. All slips from each ward were put in the opaque box separately, one ward at a time (medical, surgical, surgical, and paediatric ward). The research assistant picked the papers from the box one by one and wrote the number appearing on the slip in order to identify the corresponding patients’ records. In each hospital, 35 patients’ records were selected from medical, 35 patients’ records from surgical and 30 patients’ records were from the paediatric ward to make a total of 100 records.

The intervention was novel and we were unsure whether we would be able to recruit HP across the different disciplines and whether there would be compliance with the study. Therefore a feasibility study was carried out at a conveniently selected fifth district hospital, similar to the settings of the hospitals in the main study. There were some modifications to the training programme including shortening the training course to a single day. However, the feedback received from the feasibility training participants on the self-designed validated form to test satisfaction was very positive. Of the 55 HP participating in the feasibility study, 53 or more agreed or strongly agreed that the training had captured their interest, had been helpful to them and was important to bring change in clinical practice and to improve their service delivery. The item with the highest frequency of strongly agree endorsements (38) was “This training will improve patient outcomes” and the item with the lowest strongly agree endorsements (20) was “This training met my expectations”.

Once access had been negotiated, the logistics of offering a one day training course in four different district hospitals were finalized. Immediately prior to the training, baseline data were collected from patients’ records by a blinded trained research assistant who was a HP. The research assistant additionally audited the records at two-month, four-month, and six-month in both groups. He was unaware to which group each hospital was assigned and had minimal contact with the HP as he only engaged in record review during each visit to the different hospitals. Due to expected redundancy of information and time constraints, demographic and health conditions information were only collected from the four month audit" from the 400 patients’ records.

A two month follow-up refresher session was performed after the initial training in the experimental group hospitals whereas the control group received no follow up session. The length of the follow-up session was about two hours. The content of this session consisted of a brief recap of ICF and Interprofessional practice (IPP), views of participants on the experience and challenges met during two months of implementation and the action to be taken in order to overcome the problems and challenges. There was no further session.

### Data analysis

The analysis was performed by IBM SPSS (version21) and STATISTICA (Version 13.2 DELL INC). Descriptive statistics were used to describe the characteristics of the health care professionals who participated in this study. The experimental and control HP groups were compared at baseline to ensure equivalence between the demographic characteristics of the participants. The Chi-square test was performed to determine whether there was an association between gender, profession, place of work and group (experimental and control). Repeated measures ANOVA was done to compare the scores across the different time periods at the four sites. The post-hoc Tukey test was also used to calculate whether there was a difference in different measures. A significance level of 0.05 was used throughout the study.

#### Sample size calculation

The sample size calculation for cluster randomised trials is more complex than for simple comparison of randomly selected individuals and in order to ensure adequate power, the effective sample size or ESS needs to be calculated. The ESS is the sample required to reach adequate power, once the design effect has been taken into account. The design effect is a measure of the correlation between subjects in each cluster. The more similar they are with regard to the outcome of interest, the greater the design effect and the smaller the ESS becomes. The design effect, or inflation factor is 1 + (*n−*1) *p*, *w*here *n* = the number per cluster and *p* or rho is the intra-cluster correlation (ICC). Killip and Mahfoud [15] reported that in human studies values of ICC rho fall between 0.01 and 0.02 and we thus used a rho of .02.

It was not clear what the impact of the training would be as this was not tested during the feasibility study, using a conservative estimate of a difference of 5% between the scores of the intervention and the control hospitals, and using the SD of 8% as found in the feasibility study, 55 records were required per group, a total of 220 records. The ESS required, once the design effect had been factored, in was 12 per cluster, i.e. 67 per cluster. We concluded that a sample of 100 per cluster would thus be adequately powered to detect the predicted difference.

### Ethics

Ethical approval was obtained from the University of Cape Town, Faculty of Health Sciences Human Research Ethics Committee (Ref: HREC/REF: 085/205) and the National Health Research Committee (Ref: NHRC/2015/PROT/016) of Rwanda. The Pan-African Clinical Trial Registry number is PACTR201604001185358. Signed informed written consent was obtained from each HP who participated. Permission was sought from the hospital authorities to access the records of discharged patients and a notice was placed in the relevant wards in every hospital for patients or caregivers to read. The notice informed the patients of the study, the possibility of their data being included in the study, the benefits and possible risk of the study, patients’ privacy, and how data would be anonymised. In addition, there was a form available in every ward to be signed by the patient or caregiver who did not want his/her record to take part in the study, and then the signed form was kept in the patient record. No patients did refuse access to their records.

## Results

A total of 203 (range 50–53 per hospital) HPs participated, drawn from a large range of disciplines. This represented 88% and 93.5% of all eligible staff in the experimental and control hospitals respectively (Table 1).

**Table 1:** Distribution of participants in four hospitals at baseline.

	Experimental hospitals		Control hospitals		Total
	Hospital A	Hospital B	Hospital C	Hospital D	
Medical doctors	2	3	3	3	11
Physiotherapists	3	2	2	2	9
Nurses	44	38	38	42	162
Social workers	2	4	4	1	10
Nutritionists	1	1	1	1	4
Mental health nurses	1	1	1	1	4
Clinical psychologists		1	1		3
**Total**	**53**	**50**	**50**	**50**	**203**

The chi-square tested revealed no significant association between gender, profession, place of work and group (intervention or control). However, the intervention group had a significantly greater number of years of experience (Intervention 10.5 years, SD = 8.6 years; Control 8.0 years, SD = 6.4; separate variances t = 2.19, p = 0.003).

### Health professional participation/retention of participants

**Fig 1:**
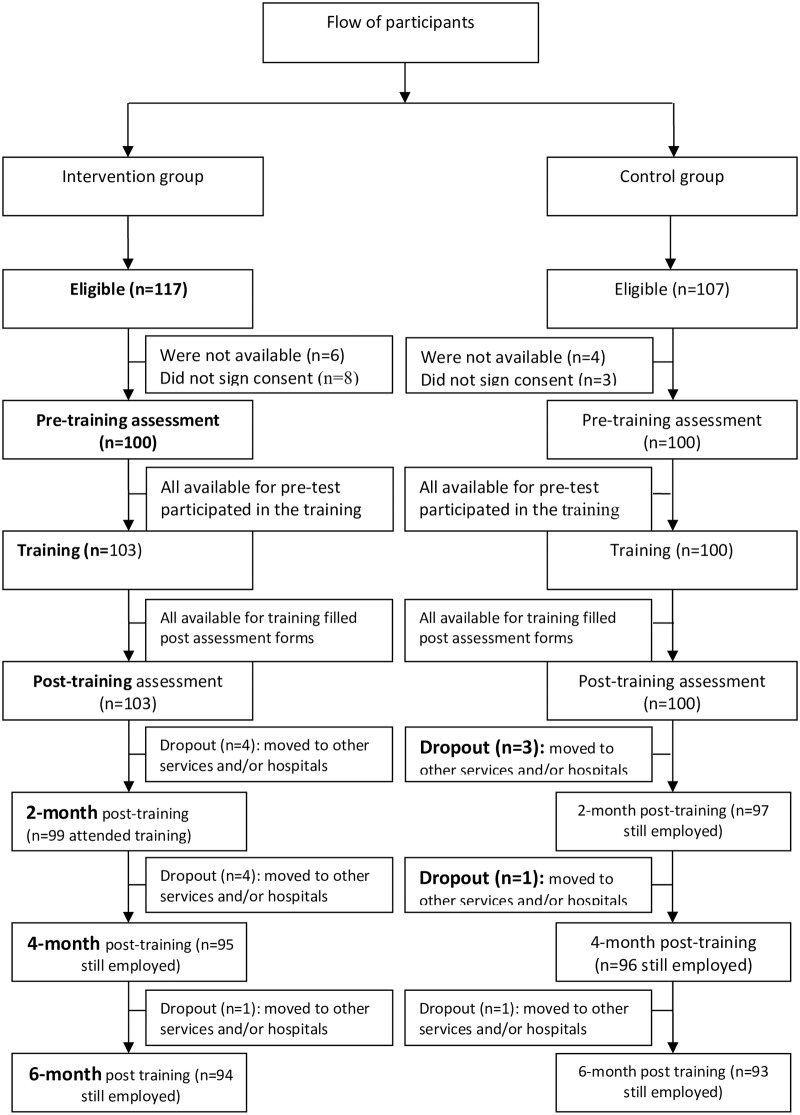
Flow chart of participants throughout the study.

The flow chart of participation below indicates that six months after the start of the trial, nine of the original group of HPs had left the employ of the experimental group and seven had left the control group hospitals. In other words, 94 of the intervention and 93 of the control group cohorts were still in the same employment six months later (Fig 1).

The demographic and medical conditions of the 400 patients whose records had been drawn were compared for the control and experimental group to test for equivalence between the two samples. The mean age of the group was 32.5 years (SD = 24.8) and there was no significant difference between the ages of the control and experimental groups (p = .828). More than 25% of both samples were under the age of ten years. There were more males in the control group (55.7%) compared to the experimental group (44.3%) and gender was significantly associated with group (Chi-sq = 7.57, p = .023). The distribution of health conditions was similar in both groups. Malaria was the most common condition (21.5% of records), followed by fractures (15%), musculoskeletal disorders (12.5%) and lower respiratory tract infections (11.25%) (Table 2).

**Table 2:** Frequency table of diagnosis/conditions at four-month.

Diagnosis/Conditions	Frequency	%
Malaria	86	21.5
Fractures	58	14.5
Gastro-intestine disorders	51	12.75
Musculoskeletal disorders	50	12.5
Lower respiratory tract	45	11.25
Wound	29	7.25
Mental disorders	27	6.75
Cardio-vascular disorders	14	3.5
Neurological condition	11	2.75
Metabolic disorders	11	2.75
HIV related conditions	10	2.5
Genito-urinary conditions	5	1.25
Upper respiratory tract	3	0.75
**Total**	**400**	**100**

Table 3 below lists the frequency and percentage of correct responses categorised by the ICF domain. At baseline, the items with the highest completion rates were those related to demographic information, such as gender and age, health conditions and impairments. Information related to activity or functional limitations, environmental factors and many of the items relating to IPP was seldom included in the notes. The difference between baseline and two months indicates that the experimental group improved in practically every domain and showed the highest difference in positive responses in the Interprofessional practice domain. The control group demonstrated a small improvement in some domains, but also a decrease in the number of patients’ records which included marital status, patient occupation and referral to other services items (Table 3).

**Table 3:** Frequency and percentage of correct responses categorised by the ICF domain.

Items		*Intervention*					*Control*				
	Information included	Baseline		Two Months		*Dif*	Baseline		Two Months		*Dif*
		N	%	N	%	*%*	N	%	N	%	*%*
Demographic information and personal factors	Patient record number	200	100	200	100	***0***	200	100	200	100	***0***
	Patient’s name	199	100	200	100	***0***	200	100	200	100	***0***
	Patient’s gender	193	97	200	100	***3***	182	91	177	89	***-2***
	Date of birth/age	199	100	200	100	***0***	181	91	182	91	***0***
	Address	198	99	200	100	***1***	175	88	182	91	***3***
	Marital status	123	88	128	91	***3***	119	85	104	74	***-11***
	Medical aid/No medical aid	185	93	186	93	***0***	164	82	170	85	***3***
	Patient occupation	89	64	118	84	***20***	96	69	81	58	***-11***
	Level of education	0	0	0	0	***0***	0	0	0	0	***0***
	Admit date	199	100	200	100	***0***	195	98	196	98	***0***
	Discharge date	182	91	200	100	***9***	192	96	199	100	***4***
	Personal factors including mental needs and spiritual	32	16	65	33	***17***	22	11	35	18	***7***
Health condition	Health condition and diagnosis	175	88	194	97	***9***	175	88	180	90	***2***
	Symptoms	167	84	181	91	***7***	161	81	171	86	***5***
Impairment	Impairment addressed	110	55	147	74	***19***	65	33	73	37	***4***
	Assessment of impairment	133	67	144	72	***5***	103	52	126	63	***11***
Activity limitation	Impact of condition on functioning	18	9	55	28	***19***	19	10	27	14	***4***
	Functioning addressed	13	7	46	23	***16***	19	10	28	14	***4***
Environmental factors (EP)	Environmental factors addressed	1	1	20	10	***9***	3	2	15	8	***6***
	Preventive measures of recurrence of health condition or complications related to condition	5	3	41	21	***18***	21	11	19	10	***-1***
	Impact of environmental factors	4	2	33	17	***15***	5	3	14	7	***4***
	Health condition managed in context	17	9	61	31	***22***	29	15	31	16	***1***
Inter-professional practice (IPP)	Referral to other services	13	7	55	28	***21***	50	25	20	10	***-15***
	Discharge note	87	44	155	78	***34***	88	44	97	49	***5***
	Referrals to other disciplines	19	10	74	37	*27*	32	16	38	19	*3*
	Case managed by different professionals	9	5	85	43	***38***	21	11	28	14	***3***
	Health professional team identified	6	3	75	38	***35***	15	8	24	12	***4***
	Health professionals treating the patient have documented	5	3	74	37	***34***	16	8	24	12	***4***

Dif: Difference

### Comparison across the four sites

**Fig 2:**
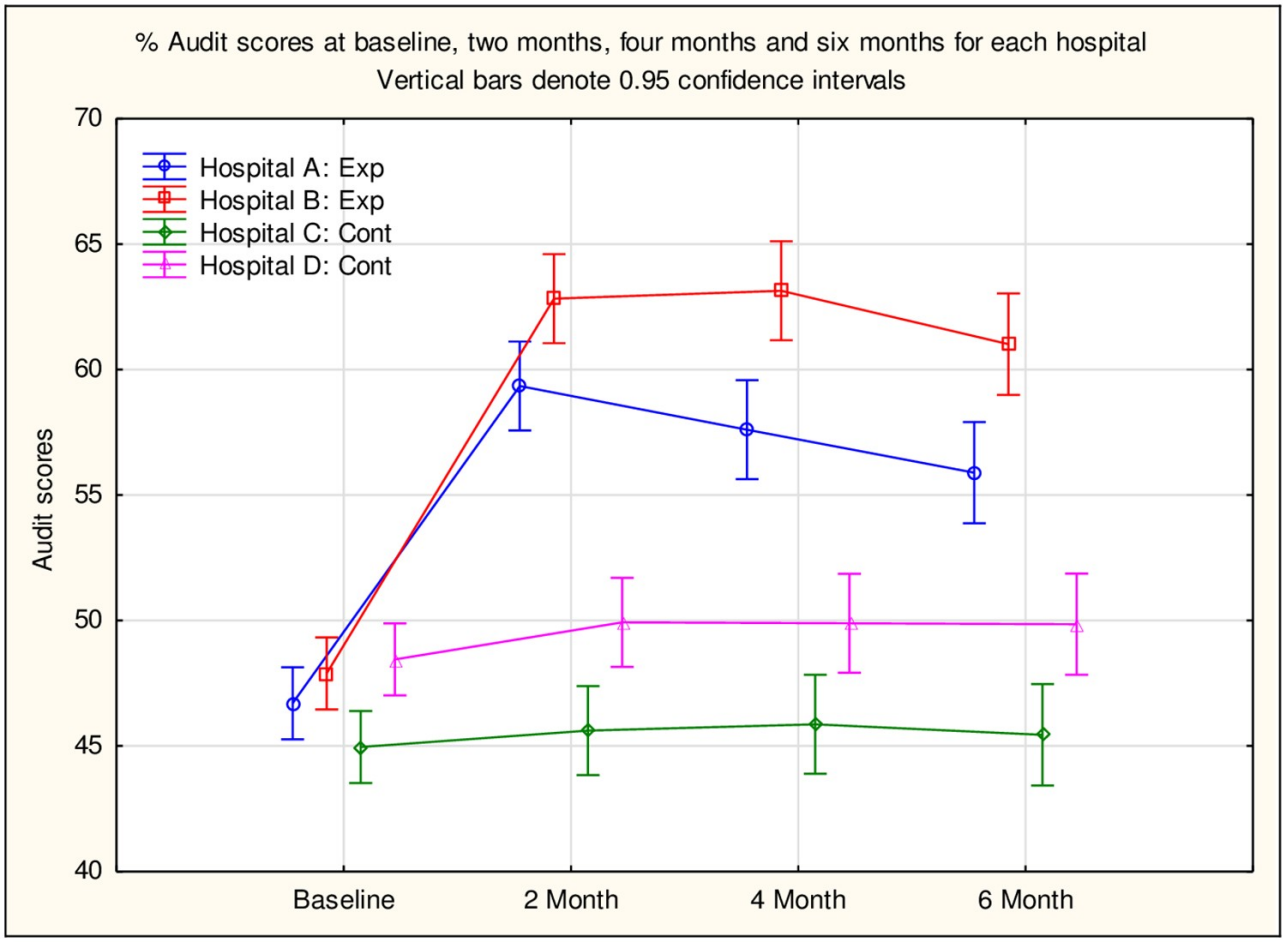
Scores across the different time periods at the four hospitals.

Repeated measures ANOVA indicated that the difference in audit scores persisted at the two intervention sites over the six month period (F (9, 1188) = 20.444, p>.001) (Fig 2).

A post hoc Tukey indicated that at baseline there was no difference between the scores at the four sites. The two control groups remained at the same level and there was no difference in their scores over time; however, from the 2-month assessment they were significantly lower than the experimental group. Although there were no significant differences between the two intervention hospitals at baseline or at 2-month assessment, Hospital A retained the improved performance better than Hospital B as there was a significant difference between the four and the six month scores.

**Fig 3:**
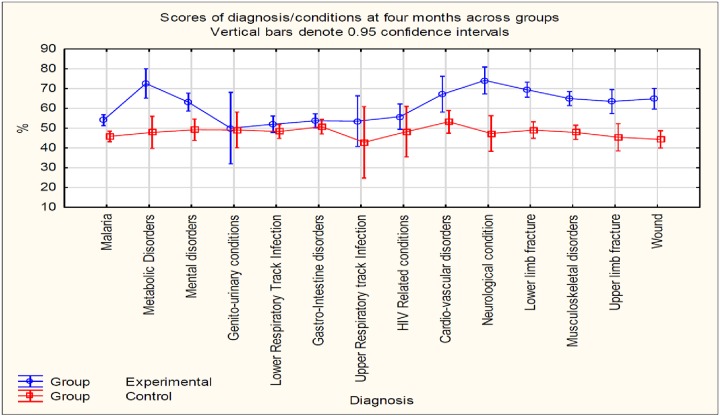
Diagnostic conditions across groups.

The health conditions were examined at four months post-intervention. The 95% confidence intervals of all conditions overlapped in the control group but not in the intervention group. The scores were significantly higher in the intervention group with the exception of acute infectious conditions such as genito-urinary and gastro-intestinal disorders, upper and lower respiratory tract infections and HIV related disorders. The largest difference between the groups was seen in metabolic and neurological conditions (Fig 3).

## Discussion

Improved behaviour in this study means improved documentation as reported by the audit of patients’ records. In summary, the experimental group hospitals showed a large improvement in the number of items documented in the records, particularly in the domain of interprofessional practice and the ICF components. This improvement was retained for six months, although the one experimental group hospital showed more decay in performance than the other. Similar information was included in the records of the control group for all conditions four months after intervention, but a differential impact of training was seen on chronic and disabling conditions in the intervention group. The difference in scores between the two groups was highest for conditions likely to need long term follow-up, such as fractures and neurological disorders.

The engagement with training exceeded our expectations with high enrolment, over 88%, for both arms of the study and representation from all disciplines. The slightly lower intervention group enrolment may be due to the increased time commitment required in this group (one day’s training as opposed to a two hour lecture). As the retention of participants throughout a longitudinal study is essential for the retention of improved behaviour [16], it was encouraging to note that only four of those who had received baseline training did not attend the two month session. The relatively low attrition over the six months (less than 8% in both experimental and control groups) is a reflection of the low staff turnover in the district hospitals and further contributed to the positive outcomes of the programme. The compliance with the programme is likely in part to reflect the weight that the endorsement of the study by the Ministry of Health and hospital authorities hold within the Rwandan health system.

With regard to the patient record samples, the homogeneity of the district hospitals ensured that the stratified random sampling resulted in two samples which were equivalent in most respects, apart from the years of experience. In fact the intervention group had a significantly greater number of years of experience which may probably influence positively or negatively the interprofessional collaboration or working together.

The sample size was adequate to detect the large differences that were apparent and considering the bed capacity, and the discharge rate at each hospital, it is likely that the current sample was an adequate representation of the patient records in the district hospitals. We concluded that the results can be generalized with confidence to other Rwandan district hospital settings but with some caution which is discussed below. However, as the training and practice of ICF and holistic care is embedded within specific contexts and health systems, the nature of training required and the impact of that training may well differ in other contexts such as central hospitals or different health systems.

### Immediate impact of training on behaviour

The primary outcome of this study was improved interprofessional practice within the district hospitals demonstrated by comprehensive patients’ records. The interprofessional collaborative practice was evidenced to be improved by effective communication. Moreover, the improved HP documentation has a positive effect to the interprofessional collaborative practice by enhancing communication [17, 18]. The baseline records were similar across the sites and supported our contention that the Bio-medical model was dominant. There was little evidence of holistic management and the information recorded was confined to basic demographic information and health condition diagnoses. No change was detected over time in the control group, although it might have been expected that, due to the Hawthorne effect, the overall completion of records might improve. This did not happen and in some items, reporting decreased. It would appear that a talk on the ICF and IPP was not sufficient to bring about behaviour change. On the other hand, the one-day training in ICF and interprofessional collaborative practice did result in a large improvement in record completion. The major flaw in the study is that it was assumed that improved documentation did reflect improved behaviour. Whereas this is a pragmatic solution to the problem of monitoring clinical performance, which may only be validly measured through observation of clinical practice, we concede that practice may have remained the same but only the documentation improved in the experimental group hospitals.

However, it appears that the documentation of all aspects of patient care did reflect plausible patterns. The items more associated with the medical model of care, i.e. demographic information such as gender, age, medical conditions and, to a lesser extent, impairments remained more or less constant or improved slightly. In addition to the underlying model of care, this is likely to be a reflection of the use of a standardised patient record which was designed to harmonise the assessment and management across the country. Demographic information and vital signs were routinely taken by nurses on admission before the patient was examined by a medical doctor, so these items might have demonstrated a ceiling effect as they were taken frequently even before intervention.

The items in which the recording showed the greatest improvement in the intervention group were related to interprofessional practice, functional limitations and environmental factors. The most improved items relating to interprofessional practice included documentation of referral to other services, discharge notes, referral to other disciplines, case managed by different professionals, and identification of the HP team treating the patient. Based on these findings, one may suggest that the interdisciplinary/interprofessional approach was infrequently used in Rwandan district hospitals. This situation was noted by Kagwiza [5] who highlighted the need to promote interprofessional collaboration based on a bio-psychosocial approach to reinforce referral within the hospital system in the Rwanda district hospitals.

The items related to activity limitation and participation restriction similarly demonstrated a considerable positive change after intervention, not only in the assessment of the impact of the health condition on functioning but also in the management of functional problems. There was also a greater awareness of environmental factor information, the need for education to prevent recurrence of health conditions and complications, and health condition management.

The positive impact of continuing professional training courses on behaviour of HPs in the workplace has been reported previously. A 34-hour training in communication skills improved nurses’ and doctors’ ability to successfully handle communication tasks that they face in their everyday practice and improved collaborative practice study [19]. Other studies have similarly reported improved interprofessional collaborative behaviour as a result of training of medical students [20] and clinical educators drawn from different disciplines [21, 22].

### Retention of desirable behaviour at 4-month and 6-month

It has been noted previously that performance in behaviour gradually decline after months following intervention [23] and we had expected to see a similar decay. However, there were differences in the rate of decay and the difference between the two intervention hospitals became more apparent. In the hospital B, the two month follow-up session may have contributed to the maintenance of the more comprehensive reporting and there was further improvement made up to four months. The hospital A started from a slightly lower base and did not make as much improvement as Hospital B. In addition, the improvement decayed more rapidly and consistently in hospital B than in Hospital A. There were similar differences in the two control group hospitals, with the one performing significantly better than the other. It seems as if the training on its own is insufficient to maintain improved behaviour and that there are contextual issues, not explored in this study, which also need to be addressed. These might include support from the management, the personal characteristics of the personnel involved (possibly medical doctors particularly), work load and general morale in the hospital. These factors should be examined in future research on the impact of training in IPP.

The retention of improvement up to six months has been reported previously by Ammentorp et al. [19] in their RCT assessing the effect of training in communication skills among nurses and medical doctors and Olsen et al. [24] in the quasi-experimental trial among physiotherapists in clinical placement. However, these studies employed questionnaires which focused on knowledge retention rather than behaviour which may be maintained for a longer period. Our study was unique in testing observable behavior and the decline noted in Hospital B indicated that behaviour may need more refresher courses and consistent field training over time.

### The use of the ICF

The conceptual framework of the ICF was found to be very useful in that it provided a model of functioning and health to introduce a holistic approach to the management of clients. The framework highlights the interrelatedness of the different aspects of the lives of patient and it assists all HP to examine the different causality paths whereby one component might influence and in turn be affected by another (e.g. functional limitation such as difficulty with walking and weight control). For example, there was an increase of 18% in reporting of measures taken to prevent complications or recurrence of the presenting health condition. The terminology is standardized and assists clear communication between the members of the IPP as the terms associated with health and functioning are well defined. This was reflected in the increased number of records in which impairments and activity limitations were assessed and subsequently addressed as separate components. Finally, as the components map to a certain extent to professional roles (e.g. social welfare officers may play an important role in addressing Environmental Factors), the framework assists the HP to delineate their specific functions and appreciate the importance of the expertise of other team members. This greater understanding of professional roles may have contributed to the improvement in the reporting of the discharge note, the referrals to other disciplines and the engagement of a team of different HP in patient management.

### Study strengths and limitations

The selection of both the districts that were to participate and the allocation to either experimental or control group used random sampling which increases the external validity of the study. In addition, a stratified random sampling was used to select patients’ records in the relevant wards in order to minimise bias and the groups were equivalent at baseline. This study used a sample which was sufficiently large to measure all the variables of interest. All measures were marked and entered by a research assistant who was blinded to group allocation. A concern was that random selection of patients’ records was not directly proportional to the total number of the available patients’ records in the ward as the number of beds in each department could not be determined and standardised prior to the study. The proportion was thus estimated based on the researcher’s experience that medical and surgical wards tend to be slightly larger than the paediatric wards.

The major weakness, as discussed above, was the assumption that documentation in the records was a valid reflection of the behaviour of the HP with regard to interprofessional teamwork and holistic care. The auditing patient record checklist used in this study was developed using a rigorous methodology and displayed excellent content validity and good internal consistency or reliability. However, the construct validity could not be tested as there was no “gold standard” by which to measure the instrument. Although it is a reasonable assumption that improved documentation reflects improved behaviour, this may not be the case. Further studies could include patient interviews or observation of clinical practice, both of which, however, could present logistical and ethical difficulties.

## Conclusions

A training programme, based on sound educational principles and using the ICF as a conceptual framework to introduce the theory and practice of IPP appeared to be effective in improving IPP in Rwandan district hospitals. Items relating to team work and increased IPP in particular were more often recorded in patient records as was information relating to the functional impact of the health condition. With the caveat that the completion of the records does reflect practice, the management of patients’ was approached in a more holistic manner after the training. It is thus suggested that the training be rolled out to district hospitals and that the ICF conceptual framework could be introduced as a model for Interprofessional Practice at training institutions. The use of a similar model should also be trialed within other contexts as it might be a cost-effective method of improving IPP and thereby improving the quality and accessibility of healthcare, based on the Lancet Commission.

## Supporting information

S1 Checklist (PDF)

S1 Protocol (PDF)

S1 Dataset (XLS)

## References

[1] World Health Organisation. How to use ICF:A Practical Manual for using the International Classification of Functioning, Disability and Health (ICF). In Geneva:WHO; 2013. http://www.who.int/classifications/drafticfpracticalmanual.pdf

[2] Frenk J, Chen L, Bhutta ZA, Cohen J, Crisp N, Evans T, et al Health professionals for a new century: Transforming education to strengthen health systems in an interdependent world. Lancet. 2010; 376(9756):1923–58. 10.1016/S0140-6736(10)61854-5 21112623

[3] van Dulmen, Lukersmith, Muxlow J, Santa Mina E, Nijhuis-van der Sanden MW van der WPG-I-NAHSG. Supporting a person-centred approach in clinical guidelines. A position paper of the Allied Health Community—Guidelines International Network (G-I-N). Heal Expect [Internet]. 2013;(iii). http://onlinelibrary.wiley.com/doi/10.1111/hex.12144/epdf 10.1111/hex.12144 PMC5060835 24118821

[4] McDougall J, Wright V, Rosenbaum P. The ICF model of functioning and disability: incorporating quality of life and human development. Dev Neurorehabil [Internet]. 2010 1 [cited 2014 Feb 6];13(3):204–11. Available from: http://www.ncbi.nlm.nih.gov/pubmed/20450470 10.3109/17518421003620525 20450470

[5] Kagwiza J. Functioning, disability and health in people living with HIV on antiretroviral therapy in Rwanda. Unpublished doctoral dissertation, University of Western Cape, South Africa. 2014.

[6] World Health Organisation. Framework for Action on Interprofessional Education & Collaborative Practice [Internet]. 2010. http://www.who.int/hrh/resources/framework_action/en/21174039

[7] Mahdizadeh M, Heydari A, Moonaghi HK. A Review of the Clinical Interdisciplinary Collaboration among Nurses and Physicians. Open J Nurs. 2015;(July):654–63.

[8] Alford VM, Remedios LJ, Webb GR, Ewen S. The use of the international classification of functioning, disability and health (ICF) in indigenous healthcare: a systematic literature review. Int J Equity Health [Internet]. 2013;12 (1):32 Available from: http://search.proquest.com/docview/1399348193?accountid=14375%5Cnhttp://rh4hh8nr6k.search.serialssolutions.com/?ctx_ver=Z39.88-2004&ctx_enc=info:ofi/enc:UTF-8&rfr_id=info:sid/ProQ:healthcompleteshell&rft_val_fmt=info:ofi/fmt:kev:mtx:journal&rft.genre=artic 23680087 10.1186/1475-9276-12-32 PMC3735045

[9] Kohler F, Connolly C, Sakaria A, Stendara K, Buhagiar M, Mojaddidi M. Can the ICF be used as a rehabilitation outcome measure? A study looking at the inter- and intra-rater reliability of ICF categories derived from an ADL assessment tool. J Rehabil Med [Internet]. 2013 9 [cited 2014 Jul 22]; 45(9):881–7. Available from: http://www.ncbi.nlm.nih.gov/pubmed/23979649 10.2340/16501977-1194 23979649

[10] Rwanda Ministry of Health. Ministry of Health (Republic of Rwanda). National Human Resources Information System (HRIS) [Internet]. 2013. http://www.moh.gov.rw/fileadmin/templates/policies/Human_Ressource_for_Health_Policy.pdf

[11] Hemming K, Girling AJ, Sitch AJ, Marsh J, Lilford RJ. Sample size calculations for cluster randomised controlled trials with a fixed number of clusters. BMC Med Res Methodol [Internet]. 2011 1 [cited 2014 Feb 18];11(1):102 Available from: http://www.pubmedcentral.nih.gov/articlerender.fcgi?artid=3149598&tool=pmcentrez&rendertype=abstract 21718530 10.1186/1471-2288-11-102 PMC3149598

[12] Grossman R, Salas E. The transfer of training: what really matters. Int J Train Dev. 2011;103–20.

[13] Baldwin T. T. and Ford JK. ‘Transfer of training: a review and directions for future research.’ Pers Psychol. 1988;41:63–105.

[14] Reeves, S., Perrier, L., Goldman, J., Freeth, D., & Zwarenstein, M. (2013). Interprofessional education: effects on professional practice and healthcare outcomes (update) (Review) SUMMARY OF FINDINGS FOR THE MAIN COMPARISON. Cochrane Database of Systematic Reviews, Art.No:CD0(3). 10.1002/14651858.CD002213.pub3.www.cochranelibrary.com PMC6513239 23543515

[15] Killip S., & Mahfound Z. (2004). What Is an Intracluster Correlation Coeffi cient? Crucial Concepts for Primary Care Researchers. *Annals of Family Medicine*, 2(3), 204–208. 10.1370/afm.141.INTRODUCTION 15209195 PMC1466680

[16] Crofts JF, Fox R, Draycott TJ, Winter C, Hunt LP, Akande VA. International Journal of Gynecology and Obstetrics Retention of factual knowledge after practical training for intrapartum emergencies. Int J Gynecol Obstet. 2013;123(1):81–5. 10.1016/j.ijgo.2013.04.015 23850035

[17] Foronda C, Macwilliams B, Mcarthur E. Nurse Education in Practice Interprofessional communication in healthcare: An integrative review. Nurse Educ Pract [Internet]. 2016;19:36–40. Available from: 10.1016/j.nepr.2016.04.005 27428690

[18] Mutshatshi TE, Mothiba TM, Mamogobo PM, Mbombi MO. Record-keeping: Challenges experienced by nurses in selected public hospitals. Curationis. 2017;1–6. 10.4102/curationis.v41i1.1931 PMC6111626 30198294

[19] Ammentorp J, Sabroe S, Kofoed P, Mainz J. The effect of training in communication skills on medical doctors ‘ and nurses ‘ self-efficacy A randomized controlled trial. Patient Educ Couns. 2007; 66:270–7. 10.1016/j.pec.2006.12.012 17337337

[20] Fernandez-Olano C, Montoya-Fernandez J, Salinas-Sanchez AS. Impact of clinical interview training on the empathy level of medical students and medical residents. Med Teach. 2008; 30:322–4. 10.1080/01421590701802299 18509879

[21] Creutzfeldt J, Hedman L, Felländer-tsai L. Effects of pre-training using serious game technology on CPR performance–an exploratory quasi-experimental transfer study. Scand J Trauma Resusc Emerg Med. 2012; 20(79):1–9. 23217084 10.1186/1757-7241-20-79 PMC3546885

[22] Fernandez-Olano C, Montoya-Fernandez J, Salinas-Sanchez AS. Impact of clinical interview training on the empathy level of medical students and medical residents. Med Teach. 2008; 30:322–4.19. 10.1080/01421590701802299 18509879

[23] Smith CJ, Peterson G, Beck GL. Handoff Training for Medical Students: Attitudes, Knowledge, and Sustainability of Skills. Educ Med J. 2015;7(2):15–26.

[24] Olsen NR, Bradley P, Espehaug B, Nortvedt MW. Evidence-Based Practice on Knowledge, Skills, Beliefs and Behaviour among Impact of a Multifaceted and Clinically Integrated Training Program in Evidence- Based Practice on Knowledge, Skills, Beliefs and Behaviour among Clinical Instructors in Physiot. 2016; (April 2015). 10.1371/journal.pone.0124332 PMC4403998 25894559

