# Supplementary material for: The impact of a training programme incorporating the conceptual framework of the International Classification of Functioning (ICF) on behaviour regarding interprofessional practice in Rwandan health professionals: A cluster randomized control trial
Source: PLoS One. 2020 Feb 7;15(2):e0226247. doi: 10.1371/journal.pone.0226247 (PMC7006896; doi:10.1371/journal.pone.0226247)
Supplement: S1 Protocol — (PDF) [file pone.0226247.s002.pdf]

Study protocol

Use of International Classification of Functioning, Disability and Health (ICF) as a Theoretical Framework to inform Interprofessional Assessment and Management by Health Care Professionals in Rwanda: A Cluster Randomised Control Trial

Author: Jean Baptiste Sagahutu (PhD student)

Supervisor: Prof Jennifer Jelsma(PhD)

Co-supervisor: A/Prof Francois Cilliers (PhD)

Local Supervisor: Dr Jeanne kagwiza (PhD)

Contents

Contents ii

List Of figures iii

List of Appendices iv

Glossary v

List of Abbreviation vii

1 Introduction 1

1.1 Background 1

1.1.1 Need for reform of medical practice 1

1.1.2 The International Classification of Functioning, Disability and Health (ICF) 2

1.1.3 Use of the ICF to improve medical care 2

1.1.4 Training of health care professionals 4

1.2 Problem Statement 5

1.3 Research questions 6

1.4 Aim and objectives of the study 6

1.4.1 Aim 6

1.4.2 Objectives 6

1.5 Justification and significance 7

2 Methodology – General 8

2.1 Research setting 8

2.2 Instrumentation 8

2.2.1 ICF Training programme 8

2.2.2 Self-designed questionnaires to determine the satisfaction with training, method of implementation and the impact of training on the knowledge of health care professionals 8

2.2.3 Auditing the quality of patient's records, referral and discharge note 9

2.2.4 Validity and Reliability of the instruments 9

3 Methodology - Feasibility study 10

3.1.1 Study design 10

3.1.2 Population and sampling 11

3.1.3 Development of intervention 11

3.1.4 Procedure 11

3.1.5 Data analysis 13

3.2 Methodology - Intervention study 14

3.2.1 Study design 14

3.2.2 Population 14

4 Implementation strategy and procedure 16

4.1 Data analysis 17

4.2 Ethical consideration 17

4.2.1 Autonomy and respect 17

4.2.2 Confidentiality 18

4.2.3 Beneficence 18

4.2.4 Justice 18

References 20

5 APPENDICES 23

List Of figures

Figure 1: The ICF framework 2

Figure 2: Feasibility study framework 10

Figure 3 : Study Conceptual Framework 19

List of Appendices

5.1 Appendix i: Study outcome framework 23

5.2 Appendix ii: Attitudes towards interprofessional team management questionnaire 24

5.3 Appendix iii: Knowledge of Health Care Professionals on Interprofessional practice and ICF . 26

5.4 Appendix iv: Health Care Professionals Satisfaction with ICF training questionnaire 29

5.5 Appendix v: Checklist for auditing patients' records to be used with ICF checklist 32

5.6 Appendix vi: International Classification of Functioning Disability and Health (ICF) checklist . 34

5.7 Appendix vii: Interprofessional care framework for continuous interprofessional care (based on ICF) 38

5.8 Appendix viii: Hospital Superintendent Information letter 39

5.9 Appendix ix: Hospital Superintendent Informed Consent form 41

5.10 Appendix x: Experimental Hospital Participant Information Letter 42

5.11 Appendix xi: Comparator Hospital Participant Information Letter 44

5.12 Appendix xii: Participant Informed consent form 46

5.13 Appendix xiii: Feasibility hospital superintendent Information Letter 47

5.14 Appendix xiv: Feasibility Hospital Participant Information Letter 49

5.15 Appendix xv: Patient Informed consent forms 51

5.16 Appendix xvi: Patient Informed Consent Form FOR MINORS to have access to the patient record 52

5.17 Appendix xvii: Parent/Guardian Informed consent form 53

Glossary

Activity limitation: Activity is the execution of a task or action by an individual**,** hence activity limitations are difficulties an individual may have in executing activities (WHO, 2002).

Biopsychosocial approach: A comprehensive model that allows people to address all major areas of the presenting issue across three spheres: physical, psychological, and sociocultural (Wade, 2009).

Environmental factors: Physical, social and attitudinal environment in which people live and conduct their life. These are factors that are not within the person's control, such as family, work, government agencies, laws, and cultural beliefs (WHO, 2002).

Health care professional: A person who by education, training, certification, or licensure is qualified to, and is engaged in, providing health care(Health Professionals Council, 2011).

Health Related Quality of life: Health-related quality of life (HRQoL) is a multi-dimensional concept that includes domains related to physical, mental, emotional, and social functioning. It goes beyond direct measures of population health, life expectancy, and causes of death, and focuses on the impact health status has on quality of life (Connor, 1993).

Holistic care: a system of comprehensive or total patient care that considers the physical, emotional, social, economic, and spiritual needs of the person; his or her response to illness; and the effect of the illness on the ability to meet self-care needs(Salas, E. and Stagl, 2009).

Impairment: Any problem in body function or structure as deviation or loss caused by physical, mental or sensory (WHO, 2002).

Interprofessional care: A group of individuals from different disciplines working and communicating with each other individuals. In the interprofessional learning environment each member provides his/her knowledge, skills, and attitudes to augment and support the contributions of others for the same management goal (Ellingson, 2002).

Participation restriction: Participation is the involvement in life situation; hence participation restrictions are problems an individual may experience in involvement in life situations (WHO, 2002).

Patient satisfaction: The degree of congruency between a patient's expectations of ideal care and his /her perception of the real care he /she receives (Ahmad & Din, 2010).

Personal factors: These are factors within the person, include race, gender, age, educational level, coping styles, etc (WHO, 2002).

Quality of life (QoL): Individual's perception of life satisfaction/quality that emerges and changes due to the impact of an individual's functioning as well as contextual factors (McDougall,et al., 2011).

***Training*:** A learning process which involves learning of new skills, concepts and behaviour (Frenk et al., 2010).

List of Abbreviation

**ICF**	International Classification of Functioning Disability and Health
**QoL**	Quality of Life
**HRQoL**	Health Related Quality of Life
**WHO**	World Health Organisation
**ICD**	International Classification of diseases
**USA**	United State of America
**CEM**	Continuous Education Meetings
**GCEHP**	Global Commission on the Education of Health professionals
**CPD**	Continuous Professional Development
**HIV/AIDs**	Human Immunodeficiency Virus Infection / Acquired Immunodeficiency Syndrome
**MOH**	Ministry of Health
**CRCT**	Cluster Randomisation Control Trial
**HCP**	Health Care Professionals
**ICC**	Intraclass correlation coefficient
**PLWH**	People living with HIV (PLWH)
**RNEC**	Rwanda National Ethics Committee
**NHRC**	National Health Research Committee

1 Introduction

There is growing criticism of the present system of health care management. This proposal presents the use of the International Classification of Functioning, Disability and Health (ICF) as a method to address the need for health care reform. The introduction section outlines the need for reform, describes the ICF and explains how it could impact on health service delivery and discusses issues related to the training of health care professionals. The problems that the thesis will address are identified and the aims and objectives of the study presented.

1.1 Background

1.1.1 Need for reform of medical practice

According to the World Health Organisation (WHO), *"health is a state of complete physical, mental and social well-being and not merely the absence of disease or infirmity"* (WHO, 1946, p.100). Despite large global gains in health status, brought about in part by the scientific application of medical science, there are still large inequities in health and health care provision, both within and between countries. Maintaining that professional education is producing graduates who are ill-equipped to deal with these problems, the Lancet commissioned a Global Independent Commission into the training of health professionals in 2010 (Frenk,et al. 2010). Problems identified by the authors included (amongst others): poor teamwork, "*persistent gender stratification of professional status" and the "so-called tribalism of the professions—i.e., the tendency of the various professions to act in isolation from or even in competition with each other*" (Frenk,et al. 2010, p.5). For example, it is estimated that a patient, during his/her period of about four days hospital stay, can be seen by around 50 different people including medical doctors, nurses, physiotherapists, and others (Daniel & Rosenstein, 2007). The lack of accurate collaboration between health care professionals can result in medical errors, lack of critical information and poor interpretation of health information, hence risks to patient's safety (Daniel & Rosenstein, 2007).

The Lancet report also identified a lack of continuity of care as impacting negatively on the health of populations. The bio-medical model of illness tends to concentrate on the health condition of the patient and treatment is provided within health institutions without taking into account the environment of the patient (Van Dulmen, et al. 2013). The bio-psychosocial model, in contrast recognises that the patient is within a certain context and that both the personal factors and environmental factors should also be considered during assessment and management (McDougall, Wright, & Rosenbaum, 2010).

1.1.2 The International Classification of Functioning, Disability and Health (ICF)

In 2001, the World Health Organisation (WHO) produced the International Classification of Functioning, Disability and Health (ICF) as a member of the International Family of Classifications and as a sister classification to the universally used International Classification of Disease (ICD). The ICD classifies health conditions such as diseases, injuries, or related states and uses an aetiological framework to gather diagnostic information.

The ICF and ICD are complementary classifications (Rauch, Cieza, & Stucki, 2008; Martinuzzi et al., 2010). The ICF conceptual framework provides a common and standardised language for the description of health and health-related states (WHO, 2002). The ICF framework does not focus only on the impairments of an individual (previously equated with disability) , but rather emphasises that functioning and disability as well as quality of life are not the linear consequence of disease or biological dysfunction (Alford, Remedios; Webb, & Ewen, 2013). Disability is therefore seen to arise as a result of the interaction between the health condition, biomedical constraints, individual and his environmental factors including the performance of activities and participation in life situation (Alford et al., 2013).

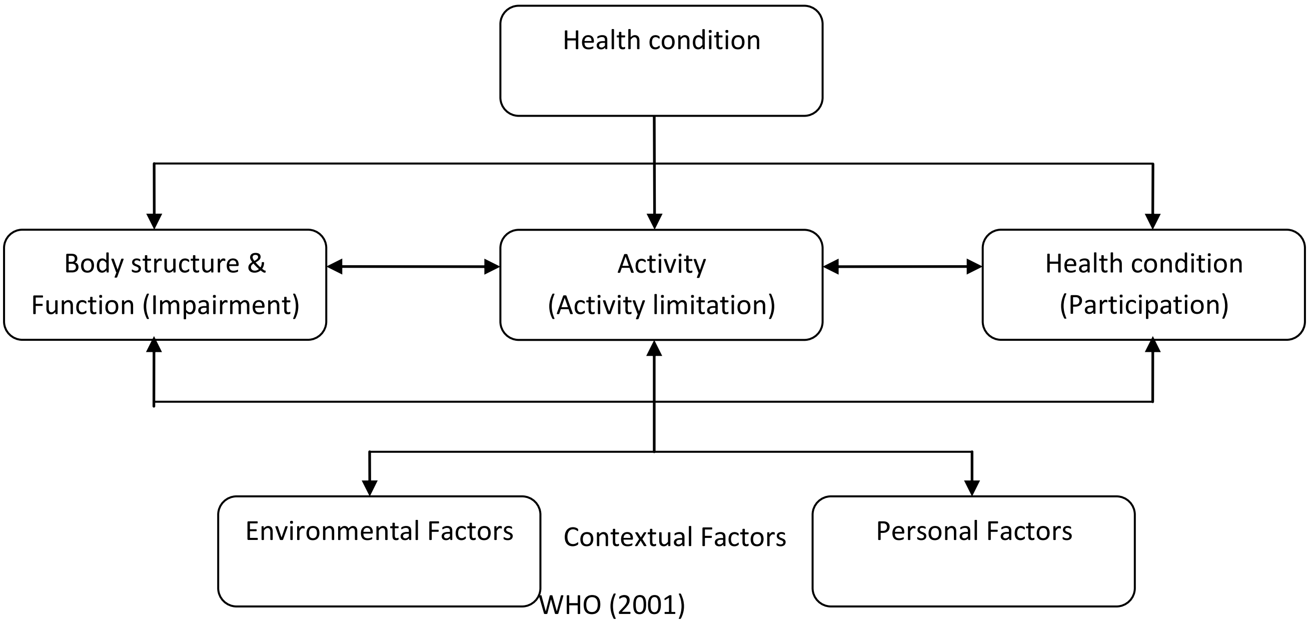

Figure 1: The ICF framework

1.1.3 Use of the ICF to improve medical care

To understand and describe the link between disease, injuries and functioning, it is recommended that health professionals should use both WHO classification systems (ICD and ICF).

Escorpizo & Bemis-Dougherty (2013) highlight the need to develop an integrated health platform which utilises information related to both the health condition/disease and the functional status of

the patient within one health information system. They maintain that the systematic capturing of the impact of disease on functioning has been hampered “by the failure to link ICD and ICF at different (conceptual and operational) levels” (Escorpizo et al., 2013).

In recognition of the need to integrate the management of functional limitation within the medical treatment of every patient, there is a process underway of harmonising the ICD and ICF in the upcoming ICD version 11. This new version will add functioning properties to the classification of disease (Escorpizo & Bemis-Dougherty, 2013). By integrating the ICF into everyday practice, health professionals, who were previously using the ICD, integrate the ICF into their everyday practice, may develop a greater understanding of the relationship between disease and functioning outcomes (Escorpizo & Bemis-Dougherty, 2013) and provide more holistic care. Furthermore, by sharing a common model of the interaction between health conditions, functioning and context such as is presented in the ICF, health professionals working in the same health setting may be better placed to work together in setting goals, evaluating treatment outcomes, and communicating as an interprofessional team with the patient and his family (WHO, 2013).

McDougall et al (2011), based on a study on the interrelationship between ICF and quality of life conceptual framework for you with chronic condition, reported that the functional and contextual factors around the health problem or disease exert a large impact on the quality of life. The same authors concluded that health services should integrate the ICF framework to include other health dimensions beyond the health condition. Similarly, the ICF was found to be a useful framework within which to assess and plan treatment by Weigl, Cieza, Kostanjsek, Kirschneck, and Stucki (2006), in their study to investigate whether ICF comprehensively covers the spectrum of health problems encountered by medical doctors and physiotherapists in patients with musculoskeletal conditions. They recommended that the ICF be integrated in clinical settings to inform an interprofessional approach within the health setting and support its validity in different professionals. A patient oriented approach is the best way of guiding interprofessional collaboration (Weigl, Cieza, Kostanjsek, Kirschneck & Stucki, 2006), but the problem highlighted by the authors is that different health professionals do not always work together. According to the researcher’s experience, this may be due to a lack of common language to be used for a smooth collaboration between professionals. To enable effective interprofessional communication to take place within the health care settings, there is a need for a comprehensive communication tool (Daniel & Rosenstein, 2007), hence the need for a training oriented towards an interprofessional collaboration.

There is evidence of the ICF being used as a measurement and assessment tool for use by physiotherapists and other rehabilitation personnel in clinics, research and teaching (Escorpizo &

Bemis-Dougherty, 2013). The ICF has been utilised across cultures in different conditions and health care settings but there is a need for further studies to examine how the ICF can guide clinical decision making across various conditions and populations (Escorpizo & Bemis-Dougherty, 2013).

The researcher's experience working in district hospitals and as a lecturer in a physiotherapy department in Rwanda leads him to conclude that the bio-medical approach is the dominant model in these clinical settings and that there is a lack of collaboration between health care professionals in different disciplines. Working in district hospital as a physiotherapist, he has encountered many different instances in which the lack of interprofessional collaboration was a barrier to a holistic approach to management.

1.1.4 Training of health care professionals

Training of health professionals is essential to bring about the desired health care reform, as they should be ensuring the universal coverage of high quality services (Frenk et al., 2010). *"Not all changes are improvements but all improvement involves change"* (Health Foundation, 2012 p.6) and health professionals need to embrace the changes required. The Global Commission on the Education of Health Professionals (GCEHP) for 21st century has reported that the biggest barrier to achieving high quality service is the application of acquired knowledge (Frenk et al., 2010). In order to address this, training and other continuous professional development (CPD) activities are crucial for both acquiring the skills to improve quality of health care and improve the motivation of health care professionals to provide excellent practice (Forsetlund et al., 2009) . It has been suggested by Anderson et al. (2011) and Health Foundation (2012) that training may enhance knowledge, skills and improve the behavior and attitudes of health professionals in their working environment. The on-going training of health care professionals can provide an important opportunity to build on the scientific foundation of health care and to provide team-based opportunity to develop collaboration between disciplines in the real world health care settings (Anderson et al., 2011).

In recent years, various training strategies have been put into practice to improve the quality of care provided by health care professionals (Blume, et al., 2010). The Continuous Professional Development (CPD) training is now mandatory in many countries to upgrade knowledge and change attitudes for the better (Khan, 2010). Although different health professional training are conducted, there is an increased emphasis on interprofessional based training, particularly in the practical based professionals (Health Foundation, 2012).

Different training methods for health care professional have been compared across the settings but the most appropriate training is still uncertain (Blume, Ford, Baldwin, & Huang, 2010). The training design and work environment have a strong relationship with transfer of training(Grossman & Salas,

2011). The active learning and participatory method which emphasises putting quality improvement into practice has been found to be effective in CPDs (Schostak ,2010). In addition, the trainees need to be facilitated to put the gained skills into practice in their working environment and the follow up sessions should also be organised for better patient outcome (Grossman & Salas, 2011). In their review on the CEM and workshops and the effects on professional practice and health care outcomes, the authors indicates the need for more studies in low and middle income countires investigating the effectiveness of training on professional quality healthcare practice and patient outcome (Forsetlund et al., 2009).

1.2 Problem Statement

The Lancet Global Independent Commission identified the need for health care reform which should be based on *"interprofessional and transprofessional education that breaks down professional silos while enhancing collaborative and non-hierarchical relationships in effective teams"* (Frenk, et al 2010, p.1950).

The need for health reform, specifically the need for an improved interprofessional collaboration, a lack of the continuum of care and over reliance on the medical model of care was noted in Rwanda, in a context similar to that of the proposed study. In a study on 500 people living with HIV (PLWH) attending district hospitals in Rwanda, Kagwiza concluded that, as the prevalence of disability in PLWH was considerable and could not be addressed simply by pharmacological medical management; there was a clear need to promote interprofessional collaboration based on a bio-psychosocial approach, to reinforce referral within the hospital system (Kagwiza, 2014). Although the subjects were all PLWH, the conclusions could be generalisable to other patient groups as well. Interprofessional patient oriented care that takes into account all factors which can determine health and functioning of an individual is thought to have the most impact on the patient's quality of life (Alford,et al., 2013).

The International Classification of Functioning Disability and Health (ICF) has been found to be a useful potential framework to help health care professionals provide a common language that looks beyond mortality and disease and instead look at how people live with their conditions(Kohler et al., 2013). This approach can provide a useful framework within which to structure the assessment of patients and other clients, not only for rehabilitation professionals but for medical practitioners and other health professionals who are involved in the care of patients (WHO, 2013). However, the ICF application is as yet somewhat limited among health professionals, especially those who are not part of rehabilitation. This is the case of health professionals of Rwanda, where the patient' assessment and management is oriented towards the condition or impairment. However, it is not clear to what

extent the ICF is known to or used by health professionals in Rwanda as a framework to inform an interprofessional patient care. Hence, there is a need to study how the ICF can be integrated in health settings in Rwanda.

1.3 Research questions

- What would be an appropriate and effective manner in which to train medical personnel in the use of the ICF to inform interprofessional practice?
- What instrumentations would be valid and reliable in terms of monitoring change in knowledge, attitudes and behaviour with regard to implementation of interprofessional practice?
- How can the ICF be used and integrated into health professionals' practice to inform interprofessional patient care in Rwanda?
- Can the use of the ICF improve the service delivery of health professionals in selected clinical settings in Rwanda?

1.4 Aim and objectives of the study

The study will consist of two phases, the first will be a feasibility study and the second a cluster randomized control study.

1.4.1 Aim

- To determine whether the ICF can be used as a framework to inform interprofessional assessment and management within hospital settings in Rwanda and whether it's use will result in improved service delivery .

1.4.2 Objectives

The specific objectives of the different phases are:

- To determine the knowledge, attitudes and behaviour of medical and allied health practitioners in Rwanda regarding interprofessional practice.
- To develop and pilot a training intervention to introduce the use of ICF into a district hospital.
- To obtain consensus from different professionals regarding the most appropriate methods to introduce the ICF conceptual framework into the routine management of patients within selected district hospitals.

- To develop and validate outcome measures that are responsive to changes in knowledge, and attitude of the medical personnel, changes in behaviour as demonstrated by improvements in patient records.

- To investigate whether a training programme on the use of the ICF in clinical practice will improve the knowledge, attitudes, and behaviour regarding interprofessional practice in selected district hospitals.

- To determine if the introduction of the ICF results in improved practice (behaviour) as seen in charged recording of patient assessment and management.

1.5 Justification and significance

In order to ensure affordable, universal health care coverage it is necessary that health care reform be undertaken. The introduction of the ICF as the framework of patient management may result in the adaptation of the bio-psycho-social model of health care and a more holistic approach to care. The patient would be managed as an individual within a context: their impairments and functional limitations will be identified and hopefully addressed, as will the environmental barriers which limit their health related quality of life. Effective collaboration between health professionals may enhance team members' awareness of each other's knowledge and skills, leading to continued improvement in management. It is anticipated that this study would come up with the recommendations of the best ways of adopting an interprofessional collaboration between health care professionals in Rwanda. Medical condition or disease alone does not predict the needed services, the hospital length of stay, the needed care as well as the patient's functional outcomes (WHO, 2002). The ICF framework has been used widely in rehabilitation medicine in high income countries, and has been found to be a very useful tool to inform a smooth collaboration between health professionals. If the use of the ICF is found to improve patient care, the training and implementation model may serve as a model for other low and middle income countries.

2 Methodology – General

2.1 Research setting

The study will be carried out in Rwanda, specifically in district hospitals. Rwanda has 40 district hospitals covering four provinces plus four district hospitals in Kigali city (Government of Rwanda, MOH, 2013).

In general, apart from Kigali city, all district hospitals in Rwanda are similar in terms of patients and conditions, services, materials and equipment as well as health personnel, so the four hospitals from Kigali will not be part of this study. The district hospitals have both inpatient and outpatient services. Outpatient services include: general consultation, dentistry, laboratory, medical imaging, mental health, ophthalmology, HIV/AIDS unit, social welfare, and physiotherapy. Inpatient services includes: orthopaedic/surgical, medical, paediatrics, and maternity. This study will only recruit patients' records from inpatient services, specifically orthopaedic/surgical, internal medicine and paediatric. This choice is based on the fact that patients on these wards may be hospitalised for longer periods and receive treatment from different health professionals than those on other wards. The maternity ward will be excluded because the mothers generally spend only one to two days in hospital.

2.2 Instrumentation

Self-designed instruments will be used for different purposes:

2.2.1 ICF Training programme

The content of the training programme will be based on literature related to best practice in continuing professional development, faculty development and transfer of training. It will also be guided by the experience of Stellenbosch University and evidence from other ICF training programs. Through the Centre for Health Professions Education, the University of Stellenbosch has experience of using ICF in interprofessional teaching and training. The major guideline of the training content is summarised in the study outcome framework (Appendix i).

2.2.2 Self-designed questionnaires to determine the satisfaction with training, method of implementation and the impact of training on the knowledge of health care professionals

Self-designed questionnaires will be developed to monitor the attitudes of the health care professionals towards interprofessional practice (Appendix ii).The questionnaire to monitor the knowledge of health professionals on interprofessional practice and ICF (Appendix iii) and questionnaire to determine the satisfaction of health care professionals with ICF training and the method of implementation (Appendix iv).

2.2.3 Auditing the quality of patient's records, referral and discharge note

A checklist will be developed and validated to determine the degree to which all aspects of the patients' health status and functioning are included in patients' records, and how referral and discharge is made. Auditing patient record checklist (Appendix v) to be used with ICF checklist (vi).

2.2.4 Validity and Reliability of the instruments

If a study will need to develop a new questionnaire, this must be piloted and validated in order to check whether it is measuring what it is supposed to measure (validity) and is it doing it consistently (reliability) (Chiaburu & Lindsay, 2008). This will be considered in this study, as it will involve developing new instruments in order to monitor appropriateness of the content, wording of questions, type and form, level of sophistication of language, sequence and how is data sought from the respondents. After development of the questionnaires, validity and reliability will be assured. A sample of 50 health care professionals and 100 patients' records will be sufficient to ensure validity and reliability of the instruments. According to the Institute of Health and Care Research (2010), a sample size of at least 50-100 participants is required to ensure the validity and reliability. The folders will be assessed until the raters of folders can do it reliably. Example: If there is 80% agreement after 20 folders, there is no need to continue. If not, the number will be increased.

2.2.4.1 Content validity and Construct validity

To ensure the content validity, the researcher will present the content of questionnaire in a panel of minimum 3 experts to review for the relevance and clarity of the items in relation to what the questionnaires are intended to measure, and also to check the flow of ideas. Content and construct validity will also be checked through cognitive debriefing by inviting a team of health care professionals to discuss on the clarity and understanding of the questions.

2.2.4.2 Reliability

To ensure the reliability of the instruments, internal consistency will be established through the use of Cronbach's Alpha as coefficient of the internal consistency for reliability test. The Cronbach's Alpha coefficient will be set at >0.70 to indicate internal consistency.

3 Methodology - Feasibility study

Prior to the main study, a feasibility study will be carried out in one selected district hospital. This is Nyamata hospital which has been selected purposively. Nyamata Hospital has the capacity of 164 beds, 14 medical doctors, 3 social workers, 79 nurses, 3 physiotherapists, and 2 nutritionists(Nyamata Hosiptal, 2012). A feasibility study is usually used to determine if the intended intervention is appropriate for further study (Taylor, Russ-Eft & Chan, 2005). This study will enable the researcher to answer the question "Can this study be done?" This study will also enable the researcher to estimate some important parameters that will be needed in the design of the main study. These could include the time needed to collect and analyse data, willingness to participate, availability and suitability of data, feasibility and duration of training, and designing a suitable outcome measure. The feasibility study will also determine if patients' records reflect the actual practice in the hospitals.

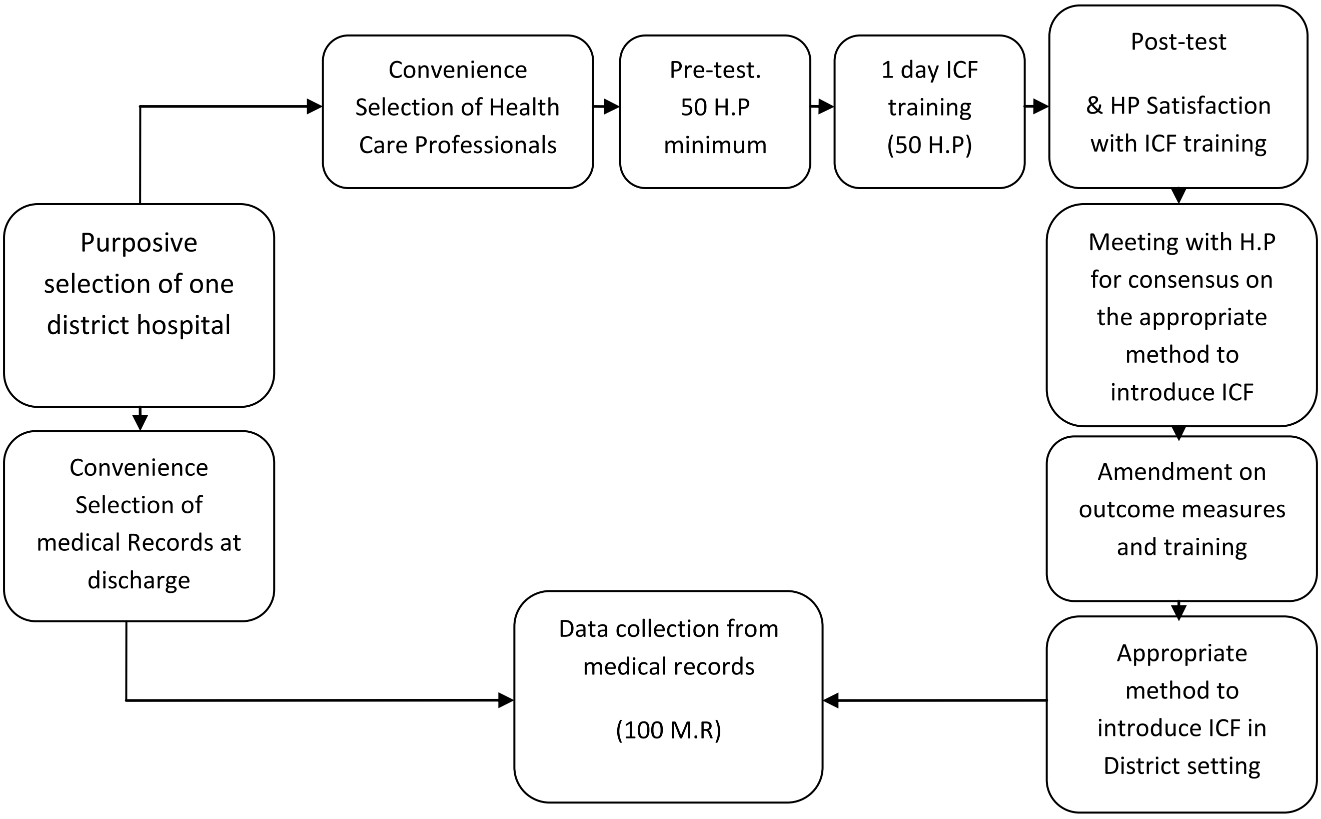

Figure 2: Feasibility study framework

3.1.1 Study design

In the feasibility study, a pre-experimental design using one group pre-test/post-test study will be used. A single selected group will be observed at two time points, once before the intervention and

once after the intervention (L.Heffner, 2004). The knowledge and attitudes of health care professionals regarding interprofessional practice will be assessed before and after a training session. The audit of medical records will also be performed. As there is no control group or randomisation to an intervention group, it is not possible to assign any change in the outcome exclusively to the intervention. However, it will give useful information with regards to planning the main experimental study.

3.1.2 Population and sampling

The population for the study will include Medical doctors (General Practitioners), Physiotherapists, Mental Health/Clinical psychologist, Nurses, Social Worker, nutritionist/dietetic working in one selected District hospital (Nyamata hospital). Patients' records for discharged patients will also be included in the study. A purposive sampling method will be used in selecting the hospital, in that the hospital authorities must be ready and willing to participate in this feasibility study. A sample of convenience will be used with regard to health professionals and patients' records under the study.

As generalisability of the results is not required, the study will use a sample of convenience and recruit all eligible staff members who wish to take part in the study; and records of 100 patients on discharge will be recruited to test the feasibility, validity and reliability of the outcome measures.

3.1.3 Development of intervention

Prior to the training, the researcher will develop the training curriculum as mentioned above. The research will present the training curriculum to the panel of experts in the ICF and training. The training curriculum will be tested as part of the feasibility study in the selected district hospital. The implementation strategy will be informed by the results from the feasibility study.

3.1.4 Procedure

The following steps will be taken in carrying out the feasibility study:

Ethical approval will be sought from the University of Cape Town Institutional Review Board. For carrying out the study in Rwanda, the approval will be sought from National Health Research Committee (NHRC), and Rwanda National Ethics Committee (RNEC). The study will also need permission from the hospital. Conveniently, the researcher will, one by one, meet the health care professional who fulfils the inclusion criteria for giving him/her a participant information letter, and those who will agree to participate will sign a written consent for agreement. This will avoid any unnecessary pressure to take part in the study. For reviewing patients' records for baseline data, the written consent will also be requested from the hospital superintendent, because patients will not be available during data collection, so permission of getting their records will be sought from the hospital superintendent. It will not be possible to obtain consent from individual patients to access

their records that have been discharged six months prior to the commencement of the study, provided that these participants will be no longer receiving care from the hospital. In their study, Zegers et al. (2007)reviewing patients records in Dutch hospital, the hospital superintendents have been consented to have access to patients records. After the intervention (training), the permission to access their folders will be requested from patients or parents/guardians. This will be done either on admission, or if not possible due to the condition of the participants, during their hospital stay. The training will be two full days and the researcher will be the one to carry out the training.

Prior to the training, the researcher will explain the title, aims, objectives, rationale and potential benefits and risks of the study. Written consent will be requested from health professionals before training. After agreement to participate in the feasibility study, the pre-test measurement will be perfumed.

Power point presentations will be used during training. The majority of medical professionals in Rwanda have been trained and use English in their daily practice. For keeping the original content of the training, the presentation will be in English and, if it will be needed, the researcher will explain in local language (Kinyarwanda) or in French for more clarity. The training will be participatory; the trainees will be allowed to raise their opinions and questions at any time. Small group discussions and case presentations will be used during the training. After a case study, the practice will be done on the patients records' for demonstration. After training, the post-measurement of knowledge and attitudes on interprofessional practice will be performed in addition with training satisfaction questionnaire.

The training will be followed by a meeting between researcher and participants. The aim of the meeting will be to reach consensus on the training content, organisation, and training procedure and materials used in the training. In addition, the most appropriate methods to introduce the ICF conceptual framework into the routine management of patients within district hospitals will be identified. The meeting will also amend the outcomes measures.

The completed questionnaires will be deposited in the provided box that will be in the safe place within the hospital. After one week the researcher will collect the questionnaires from the box. The researcher will be also collecting discharge data from the medical records in orthopaedic/surgical, medical and paediatric wards. After collecting data, the researcher will organise the data for entry in the computer. After data entry and cleaning, the researcher will do data analysis.

Once the feasibility study has been conducted and the intervention has been finalised, the details of the intervention and amended informed consent documents will be submitted as an amendment for approval to the Faculty of Health Sciences Human Research Ethics Committee (HREC).

3.1.5 Data analysis

Data analysis will be performed using STATISTICA. Descriptive statistics will be used to describe the characteristics of health professionals, such as frequency and percentage, mean and standard deviation. Within-group analysis will be used. The dependent t-Test will be used to compare the means before and after intervention (training) to detect whether there are any statistically significant differences between two means. Data will be presented in forms of tables, Scatterplots, and graphs. The ICC for absolute agreement between the two raters' scores of the audit of patient records will be used to determine the reliability of the scoring. The accuracy of interpretation of folders will be indicated by a set of ten folders of patients discharged longer than 6 months ago, assessed by researcher and research assistant (s), Data entered and compared, discussion and consensus reached. A further 10 files assessed and this process will continue until 80% agreement is reached.

3.2 Methodology - Intervention study

3.2.1 Study design

A Cluster Randomised Control Trial (CRCT) using a pragmatic study design will be used in this study. Cluster randomised controlled trials (CRCTs), is a design used in health research services whereby clusters are randomised to intervention groups. This design is primarily used to avoid contamination between the control and experimental groups when a single setting (such as a ward) is utilised (Hemming, Girling, Sitch, Marsh, & Lilford, 2011). This design will be appropriate to compare the outcome of the intervention between the experimental and comparator group.

3.2.2 Population

The population for the study will be the same as feasibility study. This will include the above mentioned health care professionals and patients' records of discharged patients. According to the reports from different hospitals, the average number of beds in each hospital is 182, Doctors (General practitioners) is 10, 80 Nurses, two Physiotherapists, one Mental Health/Clinical Psychologist, and one social worker. In general, these are the health professionals that may be found in District hospitals of Rwanda who have responsibility, at some extent, to provide services on the patients in above mentioned wards.

3.2.2.1 Inclusion criteria

Health professionals

-Full time health professionals during the period of intervention and data collection.

-Qualified health care professional.

-Health care professional working either in Orthopaedic/surgical, medical and paediatric wards.

-Health care professional with at least 6 moths of working experience in the above mentioned wards.

Patients' records

-Medical records of patients who have been admitted to the orthopedic/surgical, medical and paediatric wards for at least five days.

-Medical records of patients who have been discharged.

3.2.2.2 Sample size calculation

It is anticipated that a 20% (8) sample of 40 district hospitals will allow the outcomes to be generalised to the other hospitals. In their study, Zegers et al. (2007)reviewing patients records in Dutch hospital, a random sample of 20% of the hospitals was selected and the results were

generalized. At each hospital all the relevant health professionals will be included in the training so the sample will be one of convenience. The primary outcome of this study will be the improved interprofessional practice within the district hospitals and comprehensiveness of the patients' records demonstrated by the scores. A sample size calculation will be done once the needed tools have been finalised and the mean and standard deviation calculated during a feasibility study. However as multi-level regression analysis will be done to account for the effect of clustering, a sample of 800 patients records (100 from each hospital) should allow for the analysis of at least 80 variables. This is a large number but, as it is anticipated that the effect size will be small due to the presence of so many confounding variables, a large sample size will be required to prevent a Type II error from occurring.

3.2.2.3 Recruitment and sampling method

District hospitals

Cluster random sampling will be used by a blinded person to select the district hospitals within 40 districts of four provinces. It is anticipated that eight district hospitals will participate in this study. The sample will include Gahini hospital, Kiziguro hospital, Nemba hospital, Rutongo hospital, Remera Rukoma hospital, Nyanza hospital, Byumba hospital, and Ruhango hospital. The same individual will randomly allocate four hospitals on the experimental arm and other four hospitals on the comparator arm. The experimental hospitals will include: Gahini hospital, Nemba hospital, Rutongo hospital, and Remera Rukoma hospital. The comparator hospital will be composed of Kiziguro hospital, Ruhango hospital, Byumba hospital, and Nyanza hospital.

Health care professionals

Convenience sampling will be used to recruit the health care professionals who will participate in this study. All medical doctors and nurses who will be working in the selected district hospital (surgical, medical and paediatric wards) during the period of the study will be recruited. The sample will be also composed of all physiotherapists, social workers, mental health/psychologists, and nutritionists/dietetics who will be working in the selected district hospitals during the period of intervention and data collection.

Medical records

A stratified sampling method will also be used to select the patients' medical records that will meet the inclusion criteria to be involved in the study. It is anticipated to have a maximum of 100 patients' records per hospital. In each hospital, 100 patients' records will be audited at each occasion: baseline, two months, four months, and six months after intervention. Only the records of

discharged patients will be audited as well the records of discharged patients during two months of data collection.

4 Implementation strategy and procedure

After selecting the hospital and allocation in either experimental or control, the meeting with the hospitals will be organised. One blinded research assistant who will be different from the one who has selected the hospitals will be trained to audit medical records of all discharged patients until a maximum of 100 records in each hospital have been audited. This person will be trained on basic ICF framework and needed information from the patients' folders, and how the records will be accessed and filled as well as the selection criteria. Research assistants will also collect data from health care professionals. The measurements will be taken at baseline (one month before training) and after training. Before training, the following measurement will be performed:

- Monitoring knowledge, attitudes and behaviour of the health care professionals towards interprofessional practice (Appendix v), holistic care (Appendix vii) and the impact of functioning and environmental context on patient well-being and quality of life (Appendix viii).
- Auditing the quality of patient's records, referral and discharge note (Appendix vi)

The researcher will again train another different research assistant who will help during training. One week after, the researcher and research assistant will plan the intervention. The training procedure will be based on that used during the feasibility study, but will be adapted as needed according to the experience from the feasibility study. After training the researcher and the trained health care professionals will hold a consensus meeting regarding implementation and how they can start introducing new forms to be used with the existing records. The control hospitals would receive no training during this time, but they will be given some basic introduction on ICF theoretical. For Experimental hospitals, two months follow-up will be done after training in order to check if there are some challenges and questions during practice. Again, the comparator hospitals will receive no follow up. Three time data collection also will be done at 2nd month, 4th month, and 6th month after training will be performed in both experimental and control.
After training, the following measurement will also be performed:

- Determining the satisfaction of health care professionals with ICF training and the method of implementation (Example: Appendix iv).
- Monitoring knowledge (Appendix iii) and attitudes (Appendix ii).

- Auditing the quality of patient's records (Appendix v).

4.1 Data analysis

Data will be entered into an Excel sheet by the researcher. For checking data entry error, ten percent of data will be double entered. The error of 5% will be set, above which the data would be double entered. Descriptive statistics will be used to describe the characteristics of the hospitals, health professionals and patients. Between-group analysis will be used to establish if the two sets of groups are equivalent at baseline and at two months, four months, and six months. Depending on the type of data and whether they are normally distributed, the chi-square, Mann-Whitney U or independent t-test will be used. Within group differences, to establish whether each group has altered over time will be done and will include McNemar's test, sign test and the dependent t-test. As cluster sampling has been done, multi-level regression analysis will be done to control for the effect of clustering. For comparison of the diagnosis categories of both groups will be performed using Chi-square test to ensure that there is no association between diagnosis and groups. If necessary, sub-group analysis will be performed post-hoc to explore the impact of diagnosis on the outcome measures. The data will be presented in forms of tables, Scatterplots, and graphs.

4.2 Ethical consideration

Before conducting this study, required permission to conduct this study will be obtained from the relevant institutions and participants. Ethical approval will be sought from the University of Cape Town Institutional Review Board. For carrying out the study in Rwanda, the approval will be sought from National Health Research Committee (NHRC), and Rwanda National Ethics Committee (RNEC). The study will also need permission from the hospitals. A signed informed written consent (Appendix xii) will be requested from each participant. For reviewing patients' records for baseline data of discharged patients, the written consent will be requested from the hospital superintendent, but after the intervention (training), the informed consent form will be requested from patients or parents/guardians for having access to the folders once he/she will be discharged and assent will be obtained from minors who have the capacity to give assent.

4.2.1 Autonomy and respect

Health care professionals and patient records will be treated as autonomous agents. Hospital superintendent information letter (Appendix vii) will be provided and their informed consent form (Appendix ix) will be requested from each hospital. Permission to access patient folders will be requested from patients or parents/guardians (Appendix xv). This will be done either on admission, or if not possible due to the condition of the participants, during their hospital stay.

Experimental hospital participant information letter (Appendix x) and comparator hospital participant information letter (Appendix xi) will be provided to all participants for them to read. A signed informed written consent (Appendix xii) will be requested from each health professional to show voluntariness and comprehension. Participation in the study will be voluntary, and health professionals will be free to withdraw from the study at any time without penalty or loss of benefits. Participants will be provided with information about the study so as to empower to make choice of participating in this study.

4.2.2 Confidentiality

Participants will be assured of confidentiality and anonymity. The codes will be used rather than the names on the health professionals' questionnaires and check list for medical records. Health care professionals, after filling the questionnaire, there will be a box provided in a safe area at every hospital to submit the questionnaires. Only the researcher had access to the collected raw data that will be kept in password protected computer files, and it will be kept in a safe area.

4.2.3 Beneficence

The potential beneficence and expected risk of the study will be well explained to the health professionals who will participate in this study. This study may help inform policy in enhancing an interprofessional practice and holistic care of patients and enhance collaboration between health care professionals. No physical, psychological, social, legal and economic risk which is expected during this study.

4.2.4 Justice

For achieving social justice, a cluster randomised control trial will be used and all experimental hospitals will be treated equally in terms intervention and data collection. If the intervention is found to be effective, similar training will be offered to those hospitals in the comparator group who wish to participate.

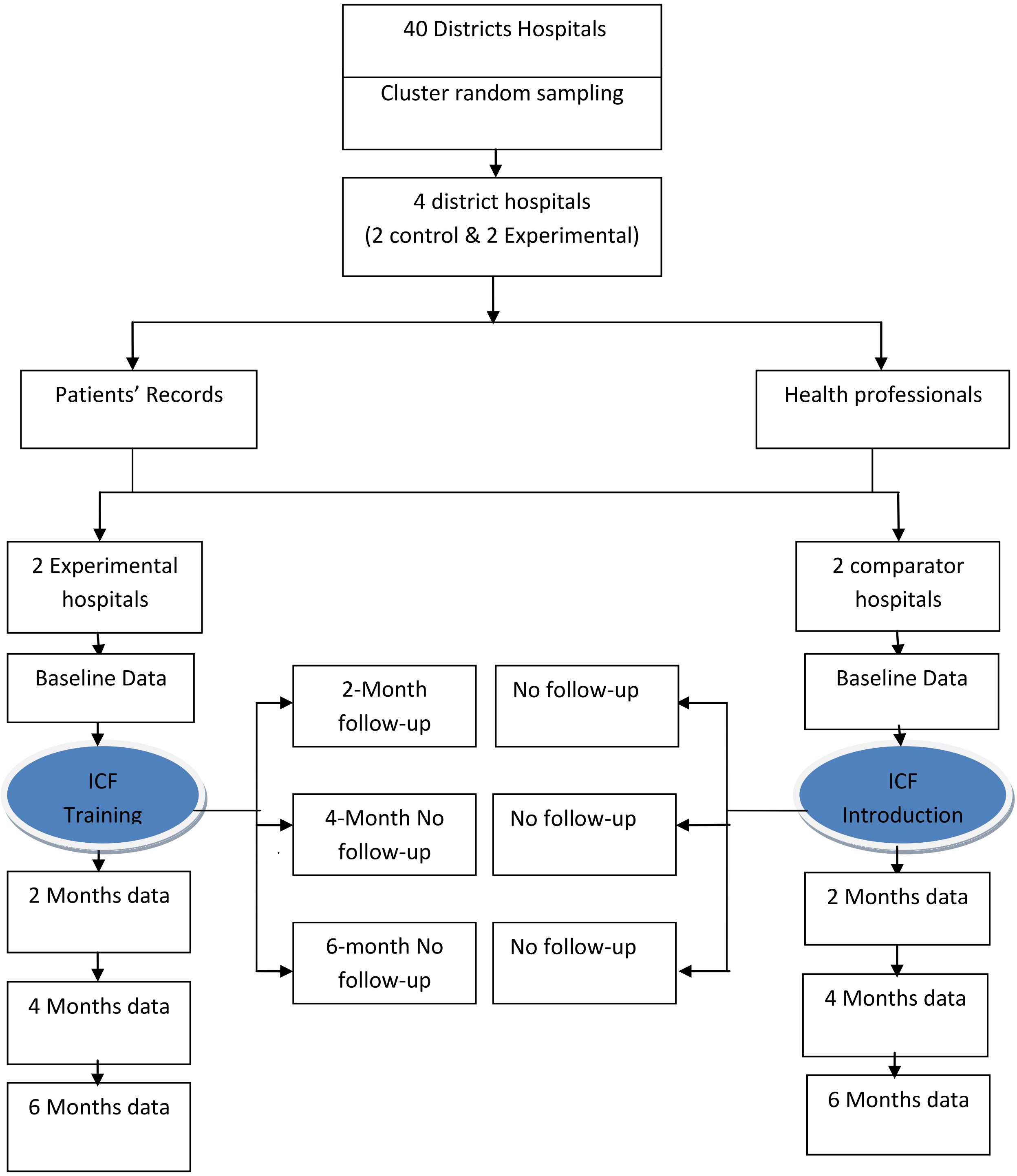

Figure 3 : Study Conceptual Framework

References

A Khan, W. (2010). Continuing Professional Development (CPD); What should we do ? Personal benefits, (01), 37–44.

Ahmad, I., & Din, S. (2010). PATIENTS ' SATISFACTION FROM THE HEALTH CARE SERVICES. *Gomal Journal of Medical Sciences*, *8*(1), 95–97.

Alford, V. M., Remedios, L. J., Webb, G. R., & Ewen, S. (2013). The use of the international classification of functioning, disability and health (ICF) in indigenous healthcare: a systematic literature review. *International Journal for Equity in Health*, *12*(1), 32. doi:10.1186/1475-9276-12-32

Anderson, C., Brock, T., Bates, I., Rouse, M., Marriott, J., Manasse, H., … Gal, D. (2011). Transforming Health Professional Education. *American Journal of Pharmaceutical Education*, *75*(2). doi:10.1016/S0140-6736(10)61854-5.

Blume, B. D., Ford, J. K., Baldwin, T. T., & Huang, J. L. (2010). Transfer of Training : A Meta-Analytic Review. *Journal of Management*, *36*(4). doi:10.1177/0149206309352880

Chiaburu, D. S. and Lindsay, D. R. (2008). (2008). "Can do or will do? The importance of self-efficacy and instrumentality for training transfer." *Human Resource Development International*, *11*, 199–206.

Connor, R. O. (1993). *ISSUES IN THE MEASUREMENT OF HEALTH- RELATED QUALITY OF LIFE*.

Daniel, M. O., & Rosenstein, A. H. (2007a). Professional Communication and Team Collaboration. *Patient Safety and Quality : An Evidence -Based Hand Book for Nurses*, *Vol.2*, 271–284.

Daniel, M. O., & Rosenstein, A. H. (2007b). Professional Communication and Team Collaboration, 1–14.

Ellingson, L. L., & Ph, D. (2002). Communication , Collaboration , and Teamwork among Health Care Professionals. *A Quarterly Review of Communication Research*, *21*(3).

Escorpizo, R., & Bemis-Dougherty, A. (2013). Introduction to Special Issue: A Review of the International Classification of Functioning, Disability and Health and Physical Therapy over the Years. *Physiotherapy Research International : The Journal for Researchers and Clinicians in Physical Therapy*, (2013). doi:10.1002/pri.1578

Escorpizo, R., Kostanjsek, N., Kennedy, C., Nicol, M. M. R., Stucki, G., & Ustün, T. B. (2013). Harmonizing WHO's International Classification of Diseases (ICD) and International Classification of Functioning, Disability and Health (ICF): importance and methods to link disease and functioning. *BMC Public Health*, *13*(1), 742. doi:10.1186/1471-2458-13-742

Forsetlund, L., Bjørndal, A., Rashidian, A., Jamtvedt, G., Ma, O. B., Wolf, F., … Ad, O. (2009). Continuing education meetings and workshops : effects on professional practice and health care outcomes (Review) SUMMARY OF FINDINGS FOR THE MAIN COMPARISON. *The Cochrane Collaboration*, (3).

Frenk. (2010). *Health Professionals for a new centry: Transforming education to strenghten health systems in an interdependent world*.

Frenk, J., Chen, L., Bhutta, Z. a, Cohen, J., Crisp, N., Evans, T., … Zurayk, H. (2010a). Health professionals for a new century: transforming education to strengthen health systems in an interdependent world. *Lancet*, *376*(9756), 1923–58. doi:10.1016/S0140-6736(10)61854-5

Frenk, J., Chen, L., Bhutta, Z. a, Cohen, J., Crisp, N., Evans, T., … Zurayk, H. (2010b). Health professionals for a new century: transforming education to strengthen health systems in an interdependent world. *Lancet*, *376*(9756), 1923–58. doi:10.1016/S0140-6736(10)61854-5

Grossman, R., & Salas, E. (2011). The transfer of training : what really matters. *International Journal of Training and Development*, 103–120.

Health Foundation. (2012). Evidence scan: Quality improvement training for healthcare professionals. *Health Foundation*, (August).

Health Professionals Council. (2011). *Professionalism in healthcare professionals*.

Hemming, K., Girling, A. J., Sitch, A. J., Marsh, J., & Lilford, R. J. (2011). Sample size calculations for cluster randomised controlled trials with a fixed number of clusters. *BMC Medical Research Methodology*, *11*(1), 102. doi:10.1186/1471-2288-11-102

Jill Schostak, Jacky Hanson, John Schostak, Tony Brown, Pter Driscoll, Ian Starke, N. J. (2010). The Effectiveness of Continuing Professional Development, 1–128.

Kagwiza, J. (2014). Functioning, disability and health in people living with HIV on antiretroviral therapy in Rwanda. Unpublished doctoral dissertation, University of Western Cape, South Africa.

Kohler, F., Connolly, C., Sakaria, A., Stendara, K., Buhagiar, M., & Mojaddidi, M. (2013). Can the ICF be used as a rehabilitation outcome measure? A study looking at the inter- and intra-rater reliability of ICF categories derived from an ADL assessment tool. *Journal of Rehabilitation Medicine*, *45*(9), 881–7. doi:10.2340/16501977-1194

L.Heffner, C. (2004). Research methods: Expermental Design.

Martinuzzi, A., Salghetti, A., Betto, S., Russo, E., Leonardi, M., Raggi, A., & Francescutti, C. (2010). The International Classification of Functioning Disability and Health, version for children and youth as a roadmap for projecting and programming rehabilitation in a neuropaediatric hospital unit. *Journal of Rehabilitation Medicine : Official Journal of the UEMS European Board of Physical and Rehabilitation Medicine*, *42*(1), 49–55. doi:10.2340/16501977-0468

McDougall, J., Wright, V., & Rosenbaum, P. (2010). The ICF model of functioning and disability: incorporating quality of life and human development. *Developmental Neurorehabilitation*, *13*(3), 204–11. doi:10.3109/17518421003620525

McDougall, J., Wright, V., Schmidt, J., Miller, L., & Lowry, K. (2011). Applying the ICF framework to study changes in quality-of-life for youth with chronic conditions. *Developmental Neurorehabilitation*, *14*(1), 41–53. doi:10.3109/17518423.2010.521795

Nyamata, Hospital (2012). REPUBLIC OF RWANDA Nyamata District Hospital Strategic Plan : 2012-2018.

Rauch, A., Cieza, A., & Stucki, G. (2008). E IN YR, *44*(3), 329–342.

Salas, E. and Stagl, K. C. (2009). Design Training Systematically and Follow the Science of Training', in E. Locke (ed.), Handbook of Principles of Organizational Behavior: Indispensible Knowledge for Evidence-Based Management, 2nd edn (Chichester: John Wiley & Sons),.

Taylor, P. J., Russ-Eft, D. F. and Chan, D. W. L. '. (2005). A meta-analytic review of behavior modeling training. *Journal of Applied Psychology*, *90*, 692–709. doi:10.1016/j.amepre.2009.02.002.How

Weigl, M., Cieza, a, Kostanjsek, N., Kirschneck, M., & Stucki, G. (2006). The ICF comprehensively covers the spectrum of health problems encountered by health professionals in patients with musculoskeletal conditions. *Rheumatology (Oxford, England)*, *45*(10), 1247–54. doi:10.1093/rheumatology/kel097

WHO, 2002. (2002). Towards a Common Language for Functioning , Disability and Health ICF. In *WHO/EIP/GPE?CAS/013*.

Zegers, M., de Bruijne, M. C., Wagner, C., Groenewegen, P. P., Waaijman, R., & van der Wal, G. (2007). Design of a retrospective patient record study on the occurrence of adverse events among patients in Dutch hospitals. *BMC Health Services Research*, *7*, 27. doi:10.1186/1472-6963-7-27

5 APPENDICES

5.1 Appendix i: Study outcome framework

Quality of in-patient patient care	Training content	Practice/ Behaviour	System changes	Knowledge	Attitudes
	Training of HCP	Patients Records	Frame-work	HCP	HCP
**Comprehensive assessment**					
Health condition and diagnosis seen in ICF context					
Personal factors including mental and spiritual needs					
Assessment of impairment					
Assessment of functioning					
Assessment of environmental factors					
**Holistic intervention**					
Health condition managed in context					
Personal factors including mental and spiritual needs					
Impairment addressed					
Functioning addressed					
Environmental factors addressed					
**Continuum of care**					
Prevention of recurrence of health condition or complications related to condition					
Referral to community services					
Liaison with community based services					
**Interprofessional practice**					
Referrals to other discipline					
Coordination of different services					
Case discussion and interprofessional treatment planning					
Integrated interprofessional ward rounds					
Task sharing and shifting					

5.2 Appendix ii: Attitudes towards interprofessional team management questionnaire

**Health professional ☑**	**Ward☑**	**Level of education☑**	**Gender ☑**
1. Medical doctor ❑	1. Medical ❑	1. A2 ❑	1.Male ❑ 2.Female ❑
2. Physiotherapist ❑	2. Surgical ❑	2. A1 ❑	
3. Nurses ❑	3.Pediatric❑	3. A0 ❑	**Age:** ………….Years
4. Social Worker ❑		4. Masters ❑	
5. Mental Health Nurse ❑		5.Other: ❑	**Years of Experience:**
6. Clinical psychologist ❑		Specify:.............	……………..Years
7. Nutritionist/Dietetic ❑			

We would like to know about your attitudes toward interprofessional health care teams and the team approach to care. By interprofessional health care team, we mean three or more health professionals (e.g., nurse, physician, social worker, physiotherapist,...) who work together and meet regularly to plan and coordinate treatment for a specific patient population. Use the scale ***SD = strongly disagree; D = disagree; N = neutral; A = agree; SA = strongly agree.*** You will mark with a symbol ☑ in appropriate box (only **one (1)** answer per statement).

**"IN MY OPINION":**	**SD**	**D**	**N**	**A**
1. Working in teams unnecessarily complicates things most of the time	❑	❑	❑	❑
2. The team approach improves the quality of care to patients	❑	❑	❑	❑
3. Team meetings foster communication among team members from different disciplines	❑	❑	❑	❑
4. Physicians have the right to alter patient care plans developed by the team	❑	❑	❑	❑
5. Patients receiving team care are more likely than other patients to be treated as whole persons	❑	❑	❑	❑
6. A team's primary purpose is to assist physicians in achieving treatment goals for patients	❑	❑	❑	❑
7. Working on a team keeps most health professionals enthusiastic and interested in their jobs	❑	❑	❑	❑
8. Patients are less satisfied with their care when it is provided by a team	❑	❑	❑	❑
9. Developing a patient care plan with other team members avoids errors in delivering care	❑	❑	❑	❑
10. When developing interprofessional patient care plans, much time is wasted translating jargon from other disciplines	❑	❑	❑	❑
11. Health professionals working on teams are more responsive than others to the emotional and financial needs of patients	❑	❑	❑	❑

12. Developing an interprofessional patient care plan is excessively time consuming	❑	❑	❑	❑
13. The physician should not always have the final word in decisions made by health care teams	❑	❑	❑	❑
14. The give and take among team members help them make better patient care decisions	❑	❑	❑	❑
15. In most instances, the time required for team meetings could be better spent in other ways	❑	❑	❑	❑
16. The physician has the ultimate legal responsibility for decisions made by the team	❑	❑	❑	❑
17. Hospital patients who receive team care are better prepared for discharge than other patients	❑	❑	❑	❑
18.Physicians are natural team leaders	❑	❑	❑	❑
19. The team approach makes the delivery of care more efficient	❑	❑	❑	❑
20. The team approach permits health professionals to meet the needs of family caregivers as well as patients	❑	❑	❑	❑
21. Having to report observations to the team helps team members better understand the work of other health professionals	❑	❑	❑	❑

Thank you very much for taking part in this research

5.3 Appendix iii: Knowledge of Health Care Professionals on Interprofessional practice and ICF

**Health professional ☑**	**Ward☑**	**Level of education☑**	**Gender ☑**
1. Medical doctor ❑	1. Medical ❑	1. A2 ❑	1.Male ❑ 2.Female ❑
2. Physiotherapist ❑	2. Surgical ❑	2. A1 ❑	
3. Nurses ❑	3.Pediatric❑	3. A0 ❑	**Age:** …………Years
4. Social Worker ❑		4. Masters ❑	
5. Mental Health Nurse ❑		5.Other: ❑	**Years of Experience:**
6. Clinical psychologist ❑		Specify:.............	……………..Years
7. Nutritionist/Dietetic ❑			

**Case study:** Mr M.G is a 45 years old man, who is overweight and has type II diabetic patient. He is receiving insulin. He was admitted to the surgical ward four weeks ago, with an open mid-shaft fracture of the right femur caused by a road traffic accident. Mr M.G is cooperative under transtibial traction and wound dressing. His occupation is a driver and he is married, with four children. Mr M.G is very concerned about the condition because he is not sure if he will go back to his job in order to support his family and to involve in church activities. The hospital has a shortage of bed adjustable available to assist him in some bed activities, consequently, due to difficulty in bed mobility, he cannot wash, dress, feed himself, and has problem with toileting. His wife is taking care of their children, so he does not have a caregiver to help in all activities of daily living which is a requirement for in-patients in hospitals. Therefore, it is difficult to get the food. Members of the community visit him once a week to help in washing, changing dress, and bringing food. Mr M.G used to smoke 20 cigarettes a day and has developed pressure sores on his buttocks, limited right leg muscle strength and knee motion, and pain on fracture site. However, he is fortunate to have a medical insurance which helps in accessing all prescribed treatment.
Based on the above case study, please complete the following table. Indicate which problems described in fall under impairments, activity limitations or participation restrictions and environmental barriers or facilitators. You should also indicate the professional who should take responsibility for managing these problems, personal and environmental factors underlying M.G problems in the 2nd column. Finally you need to describe the probable management of this problem in the 3rd column.

**Health condition**	**Profession**	**Management**
**Impairment**	**Profession**	**Management**
**Activity limitation (Current problems)**	**Profession**	**Management**
Activity limitation (Problems anticipated on discharge)	Profession	Management
**Participation restriction**	**Profession**	**Management**

**Personal factors (Positive)**	**Profession**	**Management**
**Personal Factors (Negative)**	**Profession**	**Management**
**Environmental Factors (Facilitators)**		
**Environmental Factors (Barriers)**	**Profession**	**Management**

Thank you very much for taking part in this research

5.4 Appendix iv: Health Care Professionals Satisfaction with ICF training questionnaire

Health professional ☑	Ward☑	Level of education☑	Gender ☑
1. Medical doctor ❑	1. Medical ❑	1. A2 ❑	1.Male ❑ 2.Female ❑
2. Physiotherapist ❑	2. Surgical ❑	2. A1 ❑	
3. Nurses ❑	3.Pediatric❑	3. A0 ❑	**Age:** …………Years
4. Social Worker ❑		4. Masters ❑	
5. Mental Health Nurse❑		5.Other: ❑	**Years of Experience:**
6. Clinical psychologist ❑		Specify:…………..	………………Years
7. Nutritionist/Dietetic ❑			

Please evaluate the training in terms of the succeeding sections. Your evaluations should reflect your experience and as honestly as possible. The purpose of the evaluation is to improve future training. Mark with a ☑ in the block that represents your opinion or feeling the best for each statement (only **one (1)** answer per statement). **Rating scale options:** ***1 = strongly disagree; 2 = disagree; 3 = unsure; 4 = agree; 5 = Strongly Agree.***

A. Overall Experience of the Programme	1	2	3	4	5
1. The purpose of the training programme was explained to me.	❑	❑	❑	❑	❑
2. The training programme captured my interest.	❑	❑	❑	❑	❑
3. The training was helpful to me.	❑	❑	❑	❑	❑
4. In general, I’m satisfied with the training.	❑	❑	❑	❑	❑
5. I will recommend someone else to this training programme.	❑	❑	❑	❑	❑

B. Content and Organisation	1	2	3	4	5
6. The content was appropriate and practical.	❑	❑	❑	❑	❑
7. It was introduced in the manner with good transitions.	❑	❑	❑	❑	❑
8. The training package was stimulating and exciting.	❑	❑	❑	❑	❑
9. The training met my expectation.	❑	❑	❑	❑	❑
10. I’ve learned something that is of value to me.	❑	❑	❑	❑	❑

C. The relevance of the training with clinical work	1	2	3	4	5
11. I will apply the gained knowledge in my clinical work.	❑	❑	❑	❑	❑
12. I expect the difference in the daily work because of this training.	❑	❑	❑	❑	❑
13. The training was important to bring change in clinical practice.	❑	❑	❑	❑	❑
14. This training will improve the patient outcome.	❑	❑	❑	❑	❑
15. This training will improve my service delivery.	❑	❑	❑	❑	❑

D. How much of the training was:

	1. Some	2. All	3. None
1. New	❑	❑	❑
2. Review	❑	❑	❑
3. Not relevant	❑	❑	❑

E. What was your most useful part of the training?

F. What was your least useful part of the training?

G. What are the facilitators to implement the knowledge from the training?

H. What are the barriers to implement the knowledge from this training?

J. Any other suggestion or comment to help us to improve the future training?

K. What, if anything, would you add to the training

Thank you very much for taking part in this research

5.5 Appendix v: Checklist for auditing patients' records to be used with ICF checklist

One form will be completed for each patient record. Please place a sign☑ in the box to indicate a positive response (only **one (1)** answer per statement).

**A. Informed consent**	**Yes**	**No**	**N/A**
1. The patient's consent is documented	❑	❑	❑
2. The patient's consent is signed	❑	❑	❑
**B. Patient's demographic information**			
3. Medical record number	❑	❑	❑
4. Patient's name	❑	❑	❑
5. Patient's gender	❑	❑	❑
6. Date of birth/ Age	❑	❑	❑
7. Address	❑	❑	❑
8. Marital status	❑	❑	❑
9. Medical aid/No medical aid	❑	❑	❑
10. Patient occupation	❑	❑	❑
11. Level of education	❑	❑	❑
12. Admit date	❑	❑	❑
13. Discharge date	❑	❑	❑
**C. Comprehensive assessment**			
14. Health condition and diagnosis	❑	❑	❑
15. Symptoms	❑	❑	❑
16. Assessment of impairment	❑	❑	❑
17. Impact of condition on functioning (Use ICF checklist. If yes, put the number of functioning addressed in the box).	☐	❑	❑
18. Impact of environmental factors (Use ICF Checklist. If yes, put the number of Environmental factors addressed in the box).	☐	❑	❑
**D. Holistic intervention**			
19. Health condition managed in context	❑	❑	❑
20. Personal factors including mental and spiritual needs	❑	❑	❑
21. Impairment addressed	❑	❑	❑
22. Functioning addressed	❑	❑	❑

23. Environmental factors addressed	❑	❑	❑
**E. Continuum of care and discharge**			
24. Preventive measures of recurrence of health condition or complications related to condition	❑	❑	❑
25. Referral to other services	❑	❑	❑
26. Discharge note	❑	❑	❑
**F. Inter-professional practice**			
27. Referrals to other disciplines	❑	❑	❑
28. Case managed by different professionals	❑	❑	❑
29. Health professional team identified	❑	❑	❑
30. Health professionals treating the patient have documented	❑	❑	❑

5.6 Appendix vi: International Classification of Functioning Disability and Health (ICF) checklist

One form will be completed for each patient record. Please place a sign☑ in the box to indicate a positive response (only **one (1)** answer per statement).

Activity Limitation & Participation Restriction		
**d1. LEARNING AND APPLYING KNOWLEDGE**	**Yes**	**No**
**d110** Watching	❑	❑
**d115** Listening	❑	❑
**d140** Learning to read	❑	❑
**d145** Learning to write	❑	❑
**d150** Learning to calculate *(arithmetic)*	❑	❑
**d175** Solving problems	❑	❑
**d2. GENERAL TASKS AND DEMANDS**		
**d210** Undertaking a single task	❑	❑
**d220** Undertaking multiple tasks	❑	❑
**d3. COMMUNICATION**		
**d310** Communicating with -- receiving -- spoken messages	❑	❑
**d315** Communicating with -- receiving -- non-verbal messages	❑	❑
**d330** Speaking	❑	❑
**d335** Producing non-verbal messages	❑	❑
**d350** Conversation	❑	❑
**d4. MOBILITY**		
**d430** Lifting and carrying objects	❑	❑
**d440** Fine hand use *(picking up, grasping)*	❑	❑
**d450** Walking	❑	❑
**d465** Moving around using equipment *(wheelchair, skates, etc.)*	❑	❑
**d470** Using transportation *(car, bus, train, plane, etc.)*	❑	
**d475** Driving (riding bicycle and *motorbike, driving car, etc.)*	❑	❑
**d5. SELF CARE**		
**d510** Washing oneself *(bathing, drying, washing hands, etc)*	❑	❑

**d520** Caring for body parts *(brushing teeth, shaving, grooming, etc.)*	❑	❑
**d530** Toileting	❑	❑
**d540** Dressing	❑	❑
**d550** Eating	❑	❑
**d560** Drinking	❑	❑
**d570** Looking after one`s health	❑	❑
**d6. DOMESTIC LIFE**		
**d620** Acquisition of goods and services *(shopping, etc.)*	❑	❑
**d630** Preparation of meals *(cooking etc.)*	❑	❑
**d640** Doing housework *(cleaning house, washing dishes laundry, ironing, etc.)*	❑	❑
**d660** Assisting others	❑	❑
**d7. INTERPERSONAL INTERACTIONS AND RELATIONSHIPS**		
**d710** Basic interpersonal interactions	❑	❑
**d720** Complex interpersonal interactions	❑	❑
**d730** Relating with strangers	❑	❑
**d740** Formal relationships	❑	❑
**d750** Informal social relationships	❑	❑
**d760** Family relationships	❑	❑
**d770** Intimate relationships	❑	❑
**d8. MAJOR LIFE AREAS**		
**d810** Informal education	❑	❑
**d820** School education	❑	❑
**d830** Higher education	❑	❑
**d850** Remunerative employment	❑	❑
**d860** Basic economic transactions	❑	❑
**d870** Economic self-sufficiency	❑	❑
**d9. COMMUNITY, SOCIAL AND CIVIC LIFE**		
**d910** Community Life	❑	❑
**d920** Recreation and leisure	❑	❑
**d930** Religion and spirituality	❑	❑

**d940** Human rights	❑	❑
**d950** Political life and citizenship	❑	❑

**Environmental Factors**		
**e1. PRODUCTS AND TECHNOLOGY**	**Yes**	**No**
**e110** For personal consumption *(food, medicines)*	❑	❑
**e115** For personal use in daily living	❑	❑
**e120** For personal indoor and outdoor mobility and transportation	❑	❑
**e125** Products for communication	❑	❑
**e150** Design, construction and building products and technology of buildings for public use	❑	❑
**e155** Design, construction and building products and technology of buildings for private use	❑	❑
**e2. NATURAL ENVIRONMENT AND HUMAN MADE CHANGES TO ENVIRONMENT**		
**e225** Climate	❑	❑
**e240** Light	❑	❑
**e250** Sound	❑	❑
**e3. SUPPORT AND RELATIONSHIPS**		
**e310** Immediate family	❑	❑
**e320** Friends	❑	❑
**e325** Acquaintances, peers, colleagues, neighbours and community members	❑	❑
**e330** People in position of authority	❑	❑
**e340** Personal care providers and personal assistants	❑	❑
**e355** Health professionals	❑	❑
**e360** Health related professionals	❑	❑
**e4. ATTITUDES**		
**e410** Individual attitudes of immediate family members	❑	❑
**e420** Individual attitudes of friends	❑	❑
**e440** Individual attitudes of personal care providers and personal assistants	❑	❑
**e450** Individual attitudes of health professionals	❑	❑
**e455** Individual attitudes of health related professionals	❑	❑

**e460** Societal attitudes	❑	❑
**e465** Social norms, practices and ideologies	❑	❑
**E5. SERVICES, SYSTEMS AND POLICIES**		
**e525** Housing services, systems and policies	❑	❑
**e535** Communication services, systems and policies	❑	❑
**e540** Transportation services, systems and policies	❑	❑
**e550** Legal services, systems and policies	❑	❑
**e570** Social security, services, systems and policies	❑	❑
**e575** General social support services, systems and policies	❑	❑
**e580** Health services, systems and policies	❑	❑
**e585** Education and training services, systems and policies	❑	❑
**e590** Labour and employment services, systems and policies	❑	❑

5.7 Appendix vii: Interprofessional care framework for continuous interprofessional care (based on ICF)

Patient sticker	Diagnosis:	
	Admission date:	
**Impairment**	**By who**	**Management**
**Activity limitation**	**By who**	**Management**
**Participation restriction**	**By who**	**Management**
**Personal factors**	**By who**	**Management**
**Positive**		
**Negative**		
**Environmental factors**	**By who**	**Management**
**Facilitator**		
**Barrier**		

5.8 Appendix viii: Hospital Superintendent Information letter

UNIVERSITY OF CAPE TOWN

FACULTY OF HEALTH SCIENCES

SCHOOL OF HEALTH AND REHABILITATION SCIENCES

Divisions of
Communication Sciences and Disorders, Nursing and Midwifery, Occupational Therapy, Physiotherapy

F45 Old Main Building, Groote Schuur Hospital, Observatory , 7925. Tel: +27 (0) 21 406 62505 Fax: +27 (0) 21 406 6323

Hospital Superintendent Information Letter

Name of the project: Use of International Classification of Functioning, Disability and Health (ICF) as a Theoretical Framework to inform Interprofessional Assessment and Management by Health Care Professionals in Rwanda: A Cluster Randomised Control Trial.

My name is Jean Baptiste Sagahutu and I am a postgraduate PhD student, in the Physiotherapy Department at the University of Cape Town, Cape Town, South Africa. I am currently conducting a study on the "**Use of the International Classification of Functioning, Disability and Health (ICF) as a Theoretical Framework to inform Interprofessional Assessment and Management by Health Care Professionals in Rwanda"**. I'm conducting this study for the requirement to fulfil a PhD degree in Physiotherapy. My supervisors are Professor J Jelsma, a member of the World Health Organisation Functional and Disability Reference Committee and Associate Professor Francois Cilliers, an expert in the training and education of health professionals. The ICF, which was developed by the World Health Organisation as a member of the Family of International Classifications, is intended to complement the universally utilised Internal Classification of Diseases. It not only provides a classification of functional ability but also a framework within which to explore the inter-relationship between the environment, the health condition and the functional abilities of patients. The introduction of the ICF, as the framework of patient management may result in the adaptation of the bio-psycho-social model of health care and a more holistic approach to care. It is anticipated that this study would come up with the recommendations of the best ways of adopting an interprofessional collaboration between health care professionals in Rwanda. This hospital is invited to participate because it fulfils the entire requirement of this study.

There will be two arms to the study. The experimental arm will receive a more comprehensive ICF training, whereas the comparator arm will receive a once off introduction to ICF. The experimental arm will receive two days training of health professionals working in district hospital which will include comprehensive assessment, holistic intervention, continuum of care, and interprofessional practice. Eight hours maximum per day will be used during training. For the comparator arm, the ICF introduction session will take only 3 hours.

A Cluster Randomised Control Trial will be used to select the hospital under study. This design is appropriate to compare the outcome of the intervention between the experimental and comparator group. A blinded person will randomly allocate the hospitals in either experimental or comparator arm. Therefore, based on random allocation, your hospital will be allocated to either the experimental or the control arm. Baseline and post test data will be collected to determine the knowledge, attitudes and behaviour of health professionals in Rwanda regarding interprofessional practice and holistic care of patients. This will take around 20 minutes to fill the questionnaires.

After one month, three months, and five months follow up will be performed for the experimental arm, but no follow up for comparator arm. Two months, four months, and six months data collection will be performed for both arms. Two hours sitting will be expected during each follow up meeting. The retrospective data will also be gathered from the discharged patients' records.

During the ICF training 5,000 FRW sitting allowance per day will be provided to those in the experimental arm. No money will be provided for the comparator arm. Participation in the study will be voluntary, and the freedom to withdraw from the study at any time without penalty or loss of benefits will be granted. All provided information will be kept private and confidential. The participant's name, the name of the hospital and hospital records of their patients will not be included in the report. All these will not be named at any stage for the purpose of confidentiality. Moreover, all the data collected will be kept in password protected computer files and the hard copies will be locked away.

There are no known risks associated with participating in this study. However, the participant reserve the right to withdraw from the study at any time and this will not have any effect on participant's every day work. If your hospital is selected to be a control hospital and the programme is found to be effective, you will have the option of requesting a similar programme to be run at your hospital.

Please read and sign the attached consent form if you agree that your hospital participate in this study. You are free to ask questions before or during the study and you will be answered. If you require further information please feel free to contact me through my contact details below.

Thank you for your consideration.

Yours sincerely

Physiotherapist and principal researcher Kigali/Rwanda

Jean Baptiste Sagahutu Tel: (250)0788800152 Email: jbsagahutu@khi.ac.rw

Supervisor
Jennifer Jelsma Tel: +270846116681 Email: jennifer.jelsma@uct.ac.za

"The UCT FHS Human Research Ethics Committee can be contacted on +27021 406 6338 in case participants have any questions regarding their rights and welfare as research subjects on the study."

5.9 Appendix ix: Hospital Superintendent Informed Consent form

UNIVERSITY OF CAPE TOWN

FACULTY OF HEALTH SCIENCES

SCHOOL OF HEALTH AND REHABILITATION SCIENCES

Divisions of
Communication Sciences and Disorders, Nursing and Midwifery, Occupational Therapy, Physiotherapy

F45 Old Main Building, Groote Schuur Hospital, Observatory , 7925. Tel: +27 (0) 21 406 62505 Fax: +27 (0) 21 406 6323

Hospital Superintendent Informed Consent Form

Name of the project: Use of International Classification of Functioning, Disability and Health (ICF) as a Theoretical Framework to inform Interprofessional Assessment and Management by Health Care Professionals in Rwanda: A Cluster Randomised Control Trial.

I ____ have read the Information Sheet. I understand what is required of me and I have had all my questions answered. I do not feel that I am forced to take part in this study and I am doing so of my own free will. I know that I can withdraw at any time if I so wish and that it will have no bad consequences for me.

Signed:

______________________________________ ___ ________________________

Participant Date and place

______________________________________ ________________________

Researcher Date and place

5.10 Appendix x: Experimental Hospital Participant Information Letter

UNIVERSITY OF CAPE TOWN

FACULTY OF HEALTH SCIENCES

SCHOOL OF HEALTH AND REHABILITATION SCIENCES

Divisions of

Communication Sciences and Disorders, Nursing and Midwifery, Occupational Therapy, Physiotherapy

F45 Old Main Building, Groote Schuur Hospital, Observatory , 7925. Tel: +27 (0) 21 406 62505 Fax: +27 (0) 21 406 6323

(Experimental)[1] Hospital Participant Information Letter

Name of the project: Use of International Classification of Functioning, Disability and Health (ICF) as a Theoretical Framework to inform Interprofessional Assessment and Management by Health Care Professionals in Rwanda: A Cluster Randomised Control Trial.

My name is Jean Baptiste Sagahutu and I am a postgraduate PhD student, in the Physiotherapy Department at the University of Cape Town, Cape Town, South Africa. I am currently conducting a study on the "**use of International Classification of Functioning, Disability and Health (ICF) as a Theoretical Framework to inform Interprofessional Assessment and Management by Health Care Professionals in Rwanda"**. I'm conducting this study for the requirement to fulfil a PhD degree in Physiotherapy. The ICF, which was developed by the World Health Organisation as a member of the Family of International Classifications, is intended to complement the universally utilised Internal Classification of Diseases. It not only provides a classification of functional ability but also a framework within which to explore the inter-relationship between the environment, the health condition and the functional abilities of patients. The introduction of the ICF as the framework of patient management may result in the adaptation of the bio-psycho-social model of health care and a more holistic approach to care. It is anticipated that this study would come up with the recommendations of the best ways of adopting an interprofessional collaboration between health care professionals in Rwanda. You are invited to participate because you fulfil the entire requirement of this study.

This study will involve two days training of health professionals working in district hospital which will include comprehensive assessment, holistic intervention, continuum of care, and interprofessional practice. Eight hours maximum per day will be used during training. A Cluster Randomised Control Trial has been used to select the hospital under study. This design is appropriate to compare the outcome of the intervention between the experimental and comparator group. A blinded person has randomly allocated the hospitals to either an experimental or comparator arm. Therefore, based on random allocation you have been allocated in **experimental arm**. Baseline and post test data will also be collected to determine the knowledge, attitudes and behaviour of health professionals in Rwanda regarding interprofessional practice and holistic care of patients, and patient records will be audited. This will take around 20 minutes to fill the questionnaires. After one month, three month, five month follow up; and two months, four months, and six months data collection will be performed. Two hours sitting will be expected during each follow up meeting. The follow up

[1] Will not be included

sessions will involve refreshing the participants and discuss on some problems encountered during the implementation.

During the training session 5,000 FRW sitting allowance per day will be provided. Participation in the study will be voluntary, and you will be free to withdraw from the study at any time without penalty or loss of benefits. Refusing to take part or withdrawing from the study will not affect your current or future employment at the hospital, or with the Health sector in Rwanda. All the information you provide will be kept private and confidential. Your name, the name of your hospital and hospital records of their patients will not be included in the report for confidentiality purpose

There are no known risks associated with participating in this study. However, you reserve the right to withdraw from the study at any time and this will not have any effect on your everyday work. There may be no direct benefit to participants taking part in this study. As this is a pragmatic study, even if you will not agree to take part of this study, the hospital records of patients that you might have treated will be included in the study as other staff members will also be treating these patients.

Please read and sign the attached consent form if you agree to participate. You are free to ask questions before or during the study and you will be answered. If you require further information please feel free to contact me through my contact details below.

Yours sincerely

Physiotherapist and principal researcher Kigali/Rwanda

Jean Baptiste Sagahutu   Tel: (250)0788800152   Email: jbsagahutu@khi.ac.rw

Supervisor

Jennifer Jelsma   Tel: +270846116681   Email: jennifer.jelsma@uct.ac.za

The UCT FHS Human Research Ethics Committee can be contacted on +27021 406 6338 in case participants have any questions regarding their rights and welfare as research subjects on the study.

5.11 Appendix xi: Comparator Hospital Participant Information Letter

UNIVERSITY OF CAPE TOWN

FACULTY OF HEALTH SCIENCES

SCHOOL OF HEALTH AND REHABILITATION SCIENCES

Divisions of
Communication Sciences and Disorders, Nursing and Midwifery, Occupational Therapy, Physiotherapy

F45 Old Main Building, Groote Schuur Hospital, Observatory , 7925. Tel: +27 (0) 21 406 62505 Fax: +27 (0) 21 406 6323

Comparator Hospital Participant Information Letter

Name of the project: Use of International Classification of Functioning, Disability and Health (ICF) as a Theoretical Framework to inform Interprofessional Assessment and Management by Health Care Professionals in Rwanda: A Cluster Randomised Control Trial.

My name is Jean Baptiste Sagahutu and I am a postgraduate PhD student, in the Physiotherapy Department at the University of Cape Town, Cape Town, South Africa. I am currently conducting a study on the "**use of International Classification of Functioning, Disability and Health (ICF) as a Theoretical Framework to inform Interprofessional Assessment and Management by Health Care Professionals in Rwanda"**. I'm conducting this study for the requirement to fulfil a PhD degree in Physiotherapy. The ICF, which was developed by the World Health Organisation as a member of the Family of International Classifications, is intended to complement the universally utilised Internal Classification of Diseases. It not only provides a classification of functional ability but also a framework within which to explore the inter-relationship between the environment, the health condition and the functional abilities of patients. The introduction of the ICF as the framework of patient management may result in the adaptation of the bio-psycho-social model of health care and a more holistic approach to care. It is anticipated that this study would come up with the recommendations of the best ways of adopting an interprofessional collaboration between health care professionals in Rwanda. This hospital is invited to participate because it fulfils the entire requirement of this study.

A Cluster Randomised Control Trial has been used to select the hospital under study. There will be two arms to the study. The experimental arm will receive a more comprehensive ICF training whereas the comparator arm will receive a once off introduction to ICF. This design is appropriate to compare the outcome of the intervention between the experimental and comparator group. A blinded person has been randomly allocate the hospitals in either experimental or comparator arm. Therefore, based on random allocation you have been allocated in **comparator arm**. Baseline data will be collected to determine the knowledge, attitudes and behaviour of health professionals in Rwanda regarding interprofessional practice and holistic care of patients. This will take around 20 minutes to fill the questionnaires. After one two months, four months, and six months data collection will be performed. The retrospective data will also be gathered from the discharged patients' records.

The ICF introduction session will take only 3 hours. No money will be provided for participating in this session, but some drinks will be provided. Participation in the study will be voluntary, and the freedom to withdraw from the study at any time without penalty or loss of benefits will be granted. Refusing to take part or withdrawing from the study will not affect your current or future employment at the hospital, or with the Health sector in Rwanda. All provided information will be kept private and confidential. Your name, the name of the hospital and hospital records of their patients will not be included in the report. All these will not be named at any stage for confidentiality purpose. Moreover, all the data collected will be kept in password protected computer files and the hard copies will be locked away.

There are no known risks associated with participating in this study. However, you reserve the right to withdraw from the study at any time and this will not have any effect on participant's every day work. There may be no direct benefit to participants taking part in this study. As this is a pragmatic study, even if you will not agree to take part of this study, the hospital records of patients that you might have treated will be included in the study as other staff members will also be treating these patients.

If the intervention is found to be effective, we will provide the intensive training to all those in the comparator groups who wish to participate.

Please read and sign the attached consent form if you agree to participate. You are free to ask questions before or during the study and you will be answered. If you require further information please feel free to contact me through my contact details below.

Yours sincerely

Physiotherapist and principal researcher Kigali/Rwanda

Jean Baptiste Sagahutu Tel: (250)0788800152 Email: jbsagahutu@khi.ac.rw

Supervisor

Jennifer Jelsma Tel: +270846116681 Email: jennifer.jelsma@uct.ac.za

The UCT FHS Human Research Ethics Committee can be contacted on +27021 406 6338 in case participants have any questions regarding their rights and welfare as research subjects on the study.

5.12 Appendix xii: Participant Informed consent form

UNIVERSITY OF CAPE TOWN

FACULTY OF HEALTH SCIENCES

SCHOOL OF HEALTH AND REHABILITATION SCIENCES

Divisions of Communication Sciences and Disorders,

Nursing and Midwifery, Occupational Therapy, Physiotherapy

F45 Old Main Building, Groote Schuur Hospital,

Observatory 7925

Tel: +27 (0) 21 406 62505

Fax: +27 (0) 21 406 6323

Participant Informed Consent Form

Name of the project: Use of International Classification of Functioning, Disability and Health (ICF) as a Theoretical Framework to inform Interprofessional Assessment and Management by Health Care Professionals in Rwanda: A Cluster Randomised Control Trial.

I ____ have read the Information Sheet. I understand what is required of me and I have had all my questions answered. I do not feel that I am forced to take part in this study and I am doing so of my own free will. I know that I can withdraw at any time if I so wish and that it will have no bad consequences for me. I know that if refuse to take part or withdraw withdrawing from the study will not affect my current or future employment at the hospital, or with the Health sector in Rwanda.

Signed:

_____________________________________ ___ _______________________

Participant Date and place

_____________________________________ _______________________

Researcher Date and place

5.13 Appendix xiii: Feasibility hospital superintendent Information Letter

UNIVERSITY OF CAPE TOWN

FACULTY OF HEALTH SCIENCES

SCHOOL OF HEALTH AND REHABILITATION SCIENCES

Divisions of
Communication Sciences and Disorders, Nursing and Midwifery, Occupational Therapy, Physiotherapy

F45 Old Main Building, Groote Schuur Hospital, Observatory, 7925. Tel: +27 (0) 21 406 62505 Fax: +27 (0) 21 406

<u>Feasibility hospital superintendent Information Letter</u>

Name of the project: Use of International Classification of Functioning, Disability and Health (ICF) as a Theoretical Framework to inform Interprofessional Assessment and Management by Health Care Professionals in Rwanda: A Cluster Randomised Control Trial.

My name is Jean Baptiste Sagahutu and I am a postgraduate PhD student, in the Physiotherapy Department at the University of Cape Town, Cape Town, South Africa. I am currently conducting a study on the "**Use of the International Classification of Functioning, Disability and Health (ICF) as a Theoretical Framework to inform Interprofessional Assessment and Management by Health Care Professionals in Rwanda"**. I'm conducting this study for the requirement to fulfil a PhD degree in Physiotherapy. My supervisors are Professor J Jelsma, a member of the World Health Organisation Functional and Disability Reference Committee and Associate Professor Francois Cilliers, an expert in the training and education of health professionals. The ICF, which was developed by the World Health Organisation as a member of the Family of International Classifications, is intended to complement the universally utilised Internal Classification of Diseases. It not only provides a classification of functional ability but also a framework within which to explore the inter-relationship between the environment, the health condition and the functional abilities of patients. The introduction of the ICF, as the framework of patient management may result in the adaptation of the bio-psycho-social model of health care and a more holistic approach to care. It is anticipated that this study would come up with the recommendations of the best ways of adopting an interprofessional collaboration between health care professionals in Rwanda. This hospital is invited to participate because it fulfils the entire requirement of this study.

This study is divided into two parts: a feasibility study and intervention study. Your hospital has been selected to conduct a feasibility study. During the feasibility study, a two days training of health professionals working in this hospital will include comprehensive assessment, holistic intervention, continuum of care, and interprofessional practice. Eight hours maximum per day will be used during training.

Baseline and post test data will be collected to determine the knowledge, attitudes and behaviour of health professionals regarding interprofessional practice and holistic care of patients. This will take around 20 minutes to fill the questionnaires. The retrospective data will also be gathered from the discharged patients' records.

During the ICF training, 5,000 FRW sitting allowance per day will be provided. Participation in the study will be voluntary, and the freedom to withdraw from the study at any time without penalty or loss of benefits will be granted. All provided information will be kept private and confidential. The participant's name, the name of the hospital and hospital records of their patients will not be included in the report. All these will not be named at any stage for the purpose of confidentiality. Moreover, all the data collected will be kept in password protected computer files and the hard copies will be locked away.

There are no known risks associated with participating in this study. However, the participant reserve the right to withdraw from the study at any time and this will not have any effect on participant's every day work.

Please read and sign the attached consent form if you agree that your hospital participate in this study. You are free to ask questions before or during the study and you will be answered. If you require further information please feel free to contact me through my contact details below.

Thank you for your consideration.

Yours sincerely

Physiotherapist and principal researcher Kigali/Rwanda

Jean Baptiste Sagahutu Tel: (250)0788800152 Email: jbsagahutu@khi.ac.rw

Supervisor

Jennifer Jelsma Tel: +270846116681 Email: jennifer.jelsma@uct.ac.za

The UCT FHS Human Research Ethics Committee can be contacted on +27021 406 6338 in case participants have any questions regarding their rights and welfare as research subjects on the study.

5.14 Appendix xiv: Feasibility Hospital Participant Information Letter

UNIVERSITY OF CAPE TOWN

FACULTY OF HEALTH SCIENCES

SCHOOL OF HEALTH AND REHABILITATION SCIENCES

Divisions of
Communication Sciences and Disorders, Nursing and Midwifery, Occupational Therapy, Physiotherapy

F45 Old Main Building, Groote Schuur Hospital, Observatory , 7925. Tel: +27 (0) 21 406 62505 Fax: +27 (0) 21 406 6323

Feasibility Hospital Participant Information Letter

Name of the project: Use of International Classification of Functioning, Disability and Health (ICF) as a Theoretical Framework to inform Interprofessional Assessment and Management by Health Care Professionals in Rwanda: A Cluster Randomised Control Trial.

My name is Jean Baptiste Sagahutu and I am a postgraduate PhD student, in the Physiotherapy Department at the University of Cape Town, Cape Town, South Africa. I am currently conducting a study on the "**use of International Classification of Functioning, Disability and Health (ICF) as a Theoretical Framework to inform Interprofessional Assessment and Management by Health Care Professionals in Rwanda**". I'm conducting this study for the requirement to fulfil a PhD degree in Physiotherapy. The ICF, which was developed by the World Health Organisation as a member of the Family of International Classifications, is intended to complement the universally utilised Internal Classification of Diseases. It not only provides a classification of functional ability but also a framework within which to explore the inter-relationship between the environment, the health condition and the functional abilities of patients. The introduction of the ICF as the framework of patient management may result in the adaptation of the bio-psycho-social model of health care and a more holistic approach to care. It is anticipated that this study would come up with the recommendations of the best ways of adopting an interprofessional collaboration between health care professionals in Rwanda. You are invited to participate because you fulfil the entire requirement of this study.

This study is divided into two parts: a feasibility study and intervention study. This hospital has been selected to conduct a feasibility study. This study will involve two days training of health professionals working in district hospital which will include comprehensive assessment, holistic intervention, continuum of care, and interprofessional practice. Eight hours maximum per day will be used during training. Baseline and post test data will also be collected to determine the knowledge, attitudes and behaviour of health professionals in Rwanda regarding interprofessional practice and holistic care of patients. This will take around 20 minutes to fill the questionnaires. During the training session, 5,000 FRW sitting allowance per day will be provided. Participation in the study will be voluntary, and you will be free to withdraw from the study at any time without penalty or loss of benefits. All the information you provide will be kept private and confidential.

Your name, the name of your hospital and hospital records of their patients will not be included in the report for confidentiality purpose

There are no known risks associated with participating in this study. There may be no direct benefit to participants taking part in this study. However, you reserve the right to withdraw from the study at any time and this will not have any effect on your everyday work.

Please read and sign the attached consent form if you agree to participate. You are free to ask questions before or during the study and you will be answered. If you require further information please feel free to contact me through my contact details below.

Yours sincerely

Physiotherapist and principal researcher Kigali/Rwanda

Jean Baptiste Sagahutu Tel: (250)0788800152 Email: jbsagahutu@khi.ac.rw

Supervisor

Jennifer Jelsma Tel: +270846116681 Email: jennifer.jelsma@uct.ac.za

The UCT FHS Human Research Ethics Committee can be contacted on +27021 406 6338 in case participants have any questions regarding their rights and welfare as research subjects on the study.

5.15 Appendix xv: Patient Informed consent forms

UNIVERSITY OF CAPE TOWN

FACULTY OF HEALTH SCIENCES

SCHOOL OF HEALTH AND REHABILITATION SCIENCES

Divisions of
Communication Sciences and Disorders, Nursing and Midwifery, Occupational Therapy, Physiotherapy
F45 Old Main Building, Groote Schuur Hospital, Observatory, 7925. Tel: +27 (0) 21 406 62505 Fax: +27 (0) 21 406 6323

<u>Patient Informed Consent Form to have access to the patient record</u>

My name is Jean Baptiste Sagahutu and I am a PhD student conducting a study on whether a different way of writing in the patient notes is useful. I and a research assistant, who is also a physiotherapist, want to look at the folders of patients' in this hospital to see how the records have been filled in. I'm asking for your permission to look at your folder, once you have been discharged, to see if the new way of writing records has been used and if it is useful. Your name will not be mentioned in this study and you are not forced to give the permission. If you refuse permission, it will not affect your treatment. There is no risk associated with auditing your record; however the benefit might be improved patents' record in this hospital. If you agree, we are asking you to tick in box using this sign (V) that says "YES" if you agree to let us see your notes or "NO" if you don't want us to look at your notes. Then you need to sign this form. It will then be put into your folder and when we want to look at the records, we will know that you have agreed to be part of the study. If you have not agreed, we will not look at your file.

YES I agree for my records to be looked at by the researcher. ☐

NO I do not want my records to be looked at by the researcher. ☐

Patient name: ____

Signed: ______________________ Date and place: _________________________

The UCT FHS Human Research Ethics Committee can be contacted on +27021 406 6338 in case participants have any questions regarding their rights and welfare as research subjects on the study.

5.16 Appendix xvi: Patient Informed Consent Form FOR MINORS to have access to the patient record

UNIVERSITY OF CAPE TOWN

FACULTY OF HEALTH SCIENCES

SCHOOL OF HEALTH AND REHABILITATION SCIENCES

Divisions of
Communication Sciences and Disorders, Nursing and Midwifery, Occupational Therapy, Physiotherapy

F45 Old Main Building, Groote Schuur Hospital, Observatory, 7925. Tel: +27 (0) 21 406 62505 Fax: +27 (0) 21 406 6323

Patient Informed Consent Form FOR MINORS to have access to the patient record

My name is Jean Baptiste Sagahutu and I am a PhD student who wants to find out if a different way of writing in the patient notes is useful. I and a research assistant, who is also a physiotherapist, want to look at the folders of patients' in this hospital to see how the records have been filled in. I'm asking for your permission to look at your folder, once you have been discharged, to see if the new way of writing records has been used and if it is useful. Your name will not be mentioned in this study and only the researcher will know that your notes were in the study. You are do not have to agree to take part. If you do not want to take part, it will not affect your treatment. If you allow us to look at your notes, you will not be harmed in anyway but you will also not get anything for taking part. If you agree, we are asking you to tick in box using this sign (V) that says "YES" if you agree to let us see your notes or "NO" if you don't want us to look at your notes. Then you need to sign this form. It will then be put into your folder and when we want to look at the records, we will know that you have agreed to be part of the study. If you have not agreed, we will not look at your file.

YES I agree for my records to be looked at by the researcher. ☐

NO I do not want my records to be looked at by the researcher. ☐

Patient name: ____

Signed: _____________________ Date and place: ________________________

The UCT FHS Human Research Ethics Committee can be contacted on +27021 406 6338 in case participants have any questions regarding their rights and welfare as research subjects on the study.

5.17 Appendix xvii: Parent/Guardian Informed consent form

UNIVERSITY OF CAPE TOWN

FACULTY OF HEALTH SCIENCES

SCHOOL OF HEALTH AND REHABILITATION SCIENCES

Divisions of

Communication Sciences and Disorders, Nursing and Midwifery, Occupational Therapy, Physiotherapy

F45 Old Main Building, Groote Schuur Hospital, Observatory, 7925. Tel: +27 (0) 21 406 62505 Fax: +27 (0) 21 406 6323

Parent/Guardian Informed Consent Form to have access to the child record

My name is Jean Baptiste Sagahutu and I am a PhD student conducting a study on whether a different way of writing in the patient notes is useful. I and a research assistant, who is also a physiotherapist, want to look at the folders of patients' in this hospital to see how the records have been filled in. I'm asking for your permission to look at your child's folder, once he/she have been discharged, to see if the new way of writing records has been used and if it is useful. Your child's name will not be mentioned in this study and you are not forced to give the permission. If you refuse permission, it will not affect your child's treatment. There is no risk associated with auditing your child's record; however the benefit might be improved patents' record in this hospital. If you agree, we are asking you to tick in box using this sign (V) that says "YES" if you agree to let us see your notes or "NO" if you don't want us to look at your child's notes. Then you need to sign this form. It will then be put into your folder and when we want to look at the records, we will know that you have agreed to be part of the study. If you have not agreed, we will not look at your child's file.

YES I agree for my child's records to be looked at by the researcher. ☐

NO I do not want my child's records to be looked at by the researcher. ☐

Parent/Guardian name: ____

Signed: _____________________ Date and place: ________________________

The UCT FHS Human Research Ethics Committee can be contacted on +27021 406 6338 in case participants have any questions regarding their rights and welfare as research subjects on the study.
